# Prevention and control of non-communicable diseases in antenatal, intrapartum, and postnatal care: a systematic scoping review of clinical practice guidelines since 2011

**DOI:** 10.1186/s12916-022-02508-9

**Published:** 2022-09-20

**Authors:** Jenny Jung, Eshreena K. Karwal, Steve McDonald, Tari Turner, Doris Chou, Joshua P. Vogel

**Affiliations:** 1 Maternal, Child and Adolescent Health Program, Burnet Institute, Melbourne, Australia; 2 School of Public Health and Preventive Medicine, Monash University, Melbourne, Australia; 3 Faculty of Medicine Nursing and Health Sciences, Monash University, Melbourne, Australia; 4 Department of Sexual and Reproductive Health and Research including UNDP/UNFPA/UNICEF/WHO/World Bank Special Programme of Research, Development and Research Training in Human Reproduction (HRP), World Health Organization, Geneva, Switzerland

**Keywords:** Pregnancy, Guidelines, Non-communicable diseases, World Health Organization

## Abstract

**Background:**

Non-communicable diseases (NCDs) are a leading cause of maternal mortality and morbidity worldwide. The World Health Organization is developing new recommendations focusing on the management of NCDs for pregnant, intrapartum, and postnatal women. Thus, to support the development of new guidelines and recommendations, we aimed to determine the availability, focus, and scope of recommendations of current guidelines for the management of NCDs during pregnancy, intrapartum, and postnatal period.

**Methods:**

PubMed, Global Index Medicus, TRIP, and Guideline International Network databases were searched on 31 May 2021, to identify any NCD-related guidelines published between 2011 and 2021 with no language or country restrictions. Websites of 165 professional organizations were also searched. Characteristics of included guidelines were analyzed, and recommendations were extracted from guidelines of five high-priority NCD conditions (diabetes, chronic hypertension, respiratory conditions, hemoglobinopathies and sickle cell disease, and mental and substance use disorders).

**Results:**

From 6026 citations and 165 websites, 405 guidelines were included of which 132 (33%) were pregnancy-specific and 285 (88%) were developed in high-income countries. Among pregnancy-specific guidelines, the most common conditions for which recommendations were provided were gestational diabetes, circulatory diseases, thyroid disorders, and hypertensive disorders of pregnancy. For the five high-priority conditions, 47 guidelines were identified which provided 1834 recommendations, largely focused on antenatal care interventions (62%) such as early detection, screening tools, pharmacological treatment, and lifestyle education. Postnatal recommendations largely covered postnatal clinical assessments, lifestyle education, and breastfeeding. Health system recommendations largely covered multidisciplinary care teams and strengthening referral pathways.

**Conclusions:**

This study provides a robust assessment of currently available guidelines and mapping of recommendations on NCD management within maternal health services, which will inform the scope of the World Health Organization’s future guideline development activities. This study identified a need to develop guidelines that consider NCDs holistically, with an integrated approach to antenatal, intrapartum, and postnatal care, and that are relevant for resource-limited contexts. Any such guidelines should consider what interventions are most essential to improving outcomes for women with NCDs and their newborns, and how variations in quality of NCD-related care can be addressed.

**Supplementary Information:**

The online version contains supplementary material available at 10.1186/s12916-022-02508-9.

## Background

Non-communicable diseases (NCDs) are a leading contributor to maternal mortality and morbidity worldwide [1]. NCDs encompass a broad range of conditions that are chronic in nature such as cardiovascular disease, chronic respiratory disease, diabetes, cancer, and mental disorders [1]. The prevalence of NCDs is rapidly increasing amongst women of reproductive age, and consequently a higher proportion of women are entering pregnancy with pre-existing conditions or associated risk factors such as obesity, physical inactivity, or unhealthy diet [2].

Pregnancy is a major life event accompanied by social, psychological, and physiological changes which increase the likelihood of developing certain NCDs, such as gestational diabetes mellitus (GDM) and mental disorders [3]. The burden and associated risks during pregnancy differ between NCD conditions. For example, GDM complicates one in six pregnancies worldwide [1], and when poorly controlled is associated with risk of fetal growth anomalies, obstructed labor, and stillbirth [2, 3]. In comparison, pre-existing cardiac conditions, such as ischemic heart disease and congenital heart disease, are relatively uncommon (affecting 1–4% of pregnant women), yet they are significant contributors to maternal mortality [4]. In light of the growing burden of NCDs as well as their diversity in management, it has become increasingly important to provide clinicians with evidence-based guidance to improve outcomes for women with NCDs and their newborns.

Clinical practice guidelines are defined by the Institute of Medicine as “statements that include recommendations, intended to optimize patient care, that are informed by a systematic review of evidence and an assessment of the benefits and harms of alternative care options” [5]. Guidelines provide recommendations for evidence-based decision-making in clinical care; these recommendations should ideally be based on a systematic assessment of available evidence. Producing evidence-based recommendations for global use is one of the World Health Organization’s (WHO) core functions. As part of their role in supporting evidence-informed clinical practice internationally, WHO is developing new recommendations focusing on management of NCDs for pregnant, intrapartum, and postnatal women. During 14–16 July 2021, WHO convened a technical consultation of independent experts to consider the scope of a future WHO guideline on management of NCDs in the context of antenatal, intrapartum, and postnatal care. From this consultation, the expert panel identified high-priority NCDs for WHO guideline development, including diabetes mellitus, chronic hypertension, respiratory conditions, hemoglobinopathies and sickle cell disease, obesity, and mental and substance use disorders. While studies have been conducted previously to identify and describe existing guidelines and recommendations on NCD-related maternity care, these studies focused on a single country [6] or intervention [7, 8], and a comprehensive review of all available guidelines has not been performed previously. Thus, to inform the development of WHO guidelines, this scoping review aimed to produce a comprehensive overview of existing guidelines for the management of NCDs in the maternity care context, and to further assess the focus and scope of recommendations provided.

## Methods

This scoping review was conducted in accordance with the Preferred Reporting Items for Systematic Reviews and Meta-Analyses Extension for Scoping Reviews guidelines (Additional file 1: Table 1) [9]. The protocol was registered with PROSPERO (CRD42021274492) and did not require ethical approval as this was a scoping review of publicly available clinical practice guidelines.

### Definitions

We adopted the Institute of Medicine’s definition for defining clinical practice guidelines (see above). WHO’s definition of a health intervention was used: “an act performed for, with or on behalf of a person or population whose purpose is to assess, improve, maintain, promote or modify health, functioning or health conditions” [10]. We used a broad definition for NCD categories identified from Global Health Estimates [11], including cardiovascular diseases, neoplasms, digestive diseases, respiratory diseases, diabetes mellitus, genitourinary diseases, neurological conditions, mental and substance use disorders, endocrine/blood/immune disorders, musculoskeletal diseases, congenital anomalies, skin diseases, and oral conditions. The list of NCD categories and their conditions are specified in the Additional file 2: Table 1. Additionally, to define antenatal, intrapartum, and postnatal care, the WHO definitions for stages of care [12] were used.

### Data sources and search strategy

With support from an information specialist, a search strategy was developed using medical subject headings and keywords to identify guidelines related to specified NCDs (Additional file 3: Tables 1-3. The search terms for guidelines were derived from a search filter developed by the Canadian Agency for Drugs and Technologies in Health [13]. We searched PubMed, Global Index Medicus, TRIP database, and Guideline International Network on 31 May 2021. In addition, we searched the websites of 137 specialist societies of obstetrics and gynecology (if available), 28 medical associations, and WHO (Additional file 4: Table 1).

### Inclusion criteria

Guidelines meeting the following criteria were included: (1) meets the definition of clinical practice guidelines by the Institute of Medicine (i.e., informed by a systematic review of evidence); (2) developed by a nationally recognized committee or a medical society for national, regional, or international use; (3) published between 1 January 2011 and 31 May 2021, and accessible in the public domain; and (4) provides recommendations related to NCDs for interventions on detection/diagnosis, clinical treatment or care, or health system management. To focus on contemporary clinical practice and minimize inclusion of outdated or superseded guidelines, we limited inclusion to guidelines published within the last 10 years. If multiple versions of the same guideline were identified, only the most recent version was included. Guidelines were excluded if the recommendations were provided for non-chronic conditions caused or aggravated by pregnancy. Guidelines that were developed for subnational, hospital, or local use were not eligible.

### Screening and identification of eligible guidelines

Screening was conducted using Covidence [14]. Two authors independently screened title and abstracts of records identified from the search using the aforementioned eligibility criteria. We retrieved the full texts for potentially eligible guidelines, which were also assessed in duplicate by two review authors against the same criteria. For guidelines published in a language other than English, Google Translate was used to assess eligibility. If researchers found Google Translate was insufficient to translate the guideline, they had an option to receive assistance from a translator or a public health researcher who was native in the language requiring translation. At all stages of screening, disagreements were resolved by discussion or through involvement of a third reviewer. For studies that could not be retrieved, a librarian was contacted to request a copy.

### Data extraction and description of interventions

Two authors independently extracted data using a standardized form which was pilot tested on a sample of 20 eligible guidelines and refined. At the guideline level, extracted data included title, authors/organization, publication year, country/region of publication, type of guideline (general or pregnancy-specific), number of pages, NCD category, and condition. We tagged guidelines as corresponding to an NCD condition if they had at least one recommendation for that condition. Included guidelines were classified into two broad areas—“pregnancy-specific” guidelines (i.e., the scope of the guideline pertained only to the management of pregnant, intrapartum, or postnatal women) or “general” guidelines (i.e., guidelines where the scope related to management of NCD conditions in adults or across multiple population groups, and which included specific sections on NCD-related antenatal, intrapartum, or postnatal care).

Preliminary review findings were presented at the WHO technical consultation, which informed the selection of high-priority conditions for guideline development (see Additional file 2: Table 1). We then identified the subset of guidelines that included recommendations pertaining to one or more of the high-priority conditions, and extracted all relevant recommendations. While obesity was identified as a high-priority condition during the technical consultation, it was not included in the original review protocol as it was considered to be a risk factor. For the other high-priority conditions, we extracted recommendations and classified them by stage of care (antenatal, intrapartum, or postnatal) and intervention type (screening/diagnosis, clinical, or health system). Any disagreements were resolved by discussion or with the involvement of a third reviewer.

### Data synthesis and analysis

Descriptive analysis was used to summarize identified guidelines by NCD category and condition, stage of care (antenatal, intrapartum, postnatal), and intervention type. For each high-priority condition, we prepared narrative summaries and overview tables which mapped the scope of all identified recommendations, and how many guidelines covered each intervention. We did not conduct critical appraisal of the evidence supporting the interventions recommended in identified guidelines, as this was not within the scope of the review.

### Patient and public involvement

Patients and public were not directly involved in this review; we used publicly available data for the analysis.

## Results

**Fig. 1:**
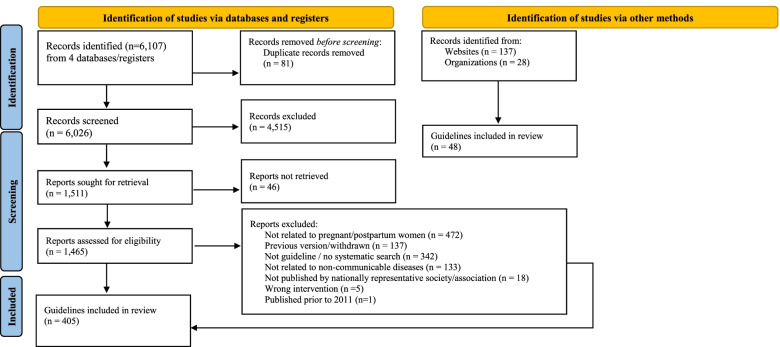
2020 PRISMA flow diagram of the search process undertaken to identify eligible guidelines, with number of records considered or excluded at each stage of the process. The database searches identified 6026 unique records, of which 357 guidelines were identified as eligible for inclusion after screening by two independent reviewers. Searches of websites of 165 organizations, societies, and colleges identified a further 48 eligible guidelines. In total, 405 guidelines were included in this review. *From:* Page MJ, McKenzie JE, Bossuyt PM, Boutron I, Hoffmann TC, Mulrow CD, et al. The PRISMA 2020 statement: an updated guideline for reporting systematic reviews. BMJ 2021;372:n71

The database searches identified 6026 unique records (PRISMA flow diagram is provided in Fig. 1). Screening of titles and abstracts identified 1511 potentially relevant records, of which 357 guidelines were identified as eligible for inclusion. Searches of websites of 165 organizations, societies, and colleges identified a further 48 eligible guidelines. In total, 405 guidelines were included in this review (Table 1).

**Table 1:** Characteristics of included clinical practice guidelines

Characteristic	Total (***n***=405)	Pregnancy-specific (***n***=132)	General (***n***=273)
**Year of publication**			
2011–2015	159	58	101
2016–2021	246	74	172
**Country of publication**^a^			
USA	88	28	60
UK	47	12	35
Australia	22	13	9
Canada	29	11	18
Italy	11	4	7
**Country-income category**^**b**^			
High-income	285	94	191
Middle-income	39	13	26
**Guideline developer**			
American medical specialty societies and organizations	67	22	45
European medical specialty societies and organizations	52	12	40
National Institute for Health and Care Excellence	11	4	7
Society of Obstetricians and Gynecologists of Canada	8	6	2
US Preventive Services Task Force	7	2	5
World Health Organization	4	3	1

^a^Five countries with highest number of guidelines published are presented only

^b^Country-income as per World Bank criteria updated for 2021 fiscal year. Clinical practice guidelines published by binational, multinational, or regional bodies not included

### Characteristics of included guidelines

**Fig. 2:**
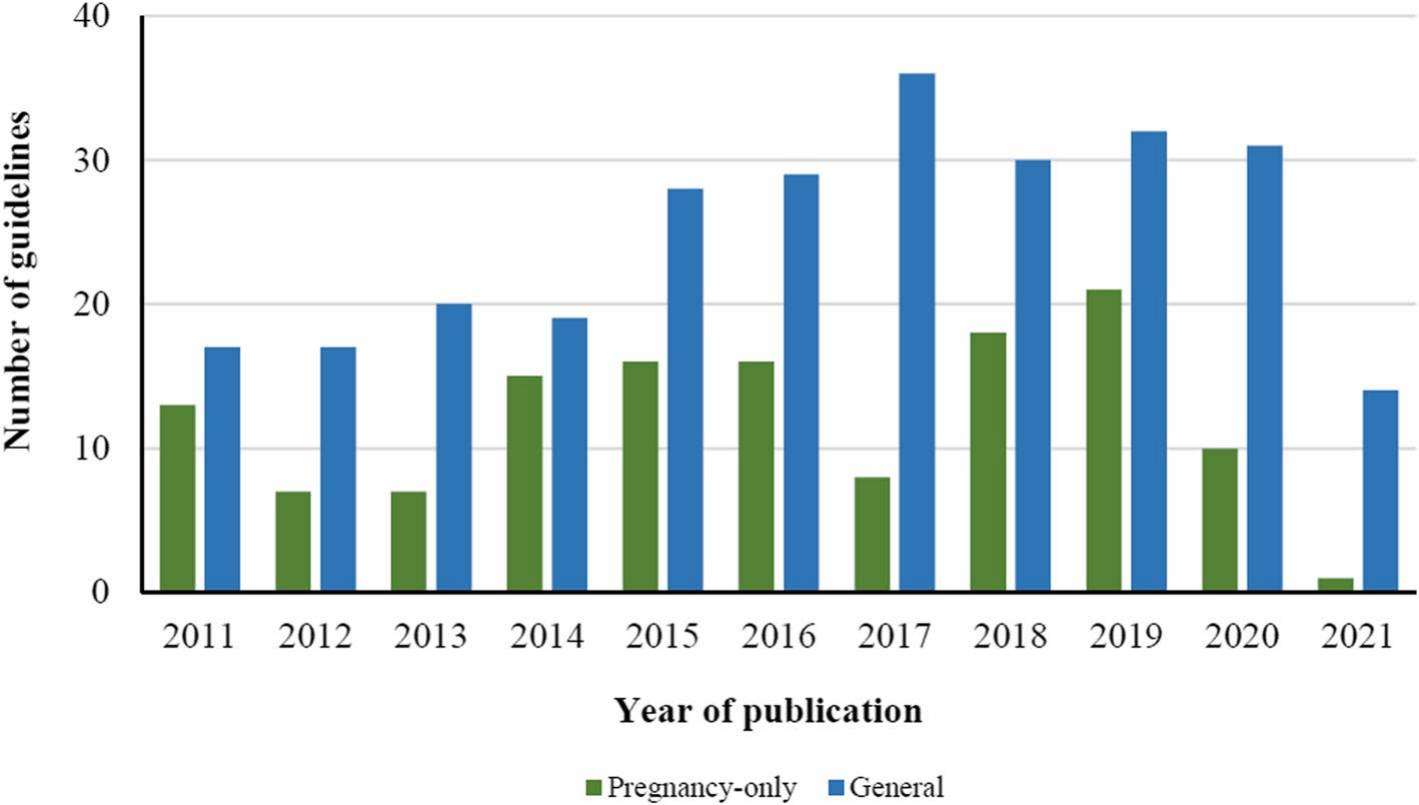
Number of general and pregnancy-specific clinical practice guidelines by year of publication. Included guidelines were classified into two broad areas—“pregnancy-specific” guidelines (i.e., the scope of the guideline pertained only to the management of pregnant, intrapartum, or postnatal women) or “general” guidelines (i.e., guidelines where the scope related to management of NCD conditions in adults or across multiple population groups, and which included specific sections on NCD-related antenatal, intrapartum, or postnatal care). In total, 246 (61%) guidelines were published between 2016 and 2021, and the highest number of guidelines published in 1 year was in 2019 (*n*=53)

A summary of the characteristics of included guidelines is provided in Table 1, and a further detailed table of characteristics is provided in Additional file 5: Table 1. Of the 405 guidelines, 132 were pregnancy-specific and 273 were general type guidelines. In total, 246 (61%) guidelines were published between 2016 and 2021, and the highest number of guidelines published in 1 year was in 2019 (*n*=53) (Fig. 2). The countries with highest number of guidelines issued were United States of America (USA) (*n*=88), United Kingdom (UK) (*n*=47), Canada (*n*=29), Australia (*n*=22), and Italy (*n*=11). The majority of guidelines were from high-income countries (HICs, *n*=285), while the remaining 39 guidelines were from middle-income countries (MICs), most commonly Brazil (*n*=9), China (*n*=6), Malaysia (*n*=6), and Thailand (*n*=3). Other middle-income countries were Georgia, India, South Africa, Argentina, Colombia, Costa Rica, Iran, Kenya, and Romania. There were no guidelines identified from low-income countries. Guidelines were published by a wide range of organizations, including medical specialty societies and organizations from USA (*n*=67), Europe (*n*=52), and the UK National Institute for Health and Care Excellence (NICE) (*n*=11).

### Distribution of guidelines by disease category and condition

**Fig. 3:**
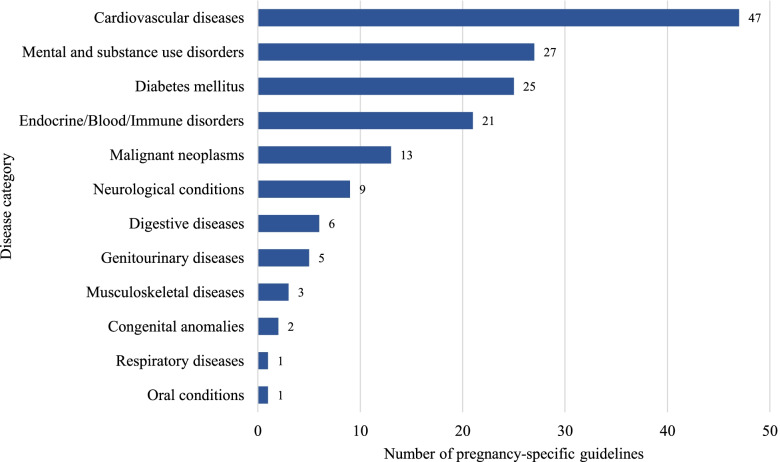
Distribution of pregnancy-specific clinical practice guidelines by disease category. Included guidelines were classified as “pregnancy-specific guidelines” if the scope of the guideline pertained only to the management of pregnant, intrapartum, or postnatal women. The most common disease categories covered by pregnancy-specific guidelines were cardiovascular diseases, mental and substance use disorders, and diabetes mellitus.

**Fig. 4:**
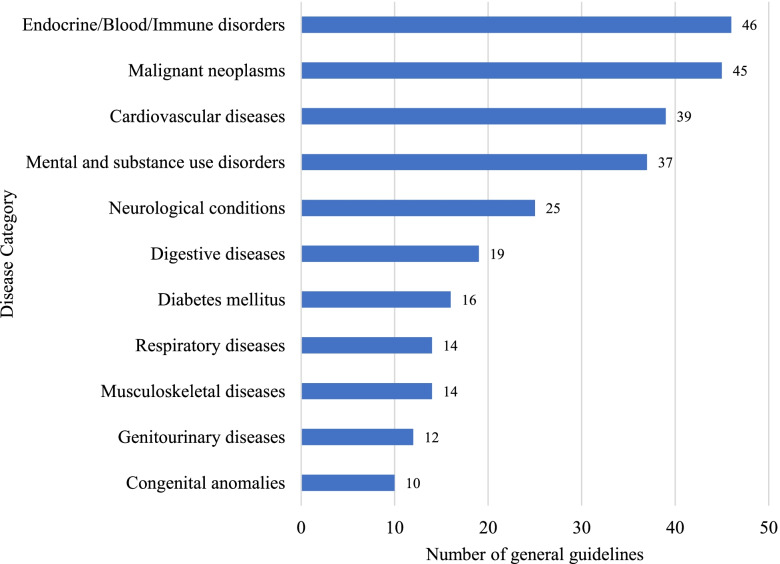
Distribution of general clinical practice guidelines by disease category. Included guidelines were classified as “general guidelines” if the scope related to management of non-communicable disease conditions in adults or across multiple population groups, and which included specific sections on non-communicable disease related antenatal, intrapartum, or postnatal care. The most common disease categories covered by general guidelines were endocrine/blood/immune disorders, malignant neoplasms, and cardiovascular diseases

The number and distribution of guidelines by disease category and condition are described for pregnancy-specific and general guidelines (Figs. 3 and 4 respectively). In pregnancy-specific guidelines, the most common conditions for which recommendations were provided were GDM, other circulatory diseases, disorders of the thyroid gland, hypertensive disorders of pregnancy, general cardiovascular diseases, and depressive disorders. For general guidelines, common conditions were other circulatory diseases, cervical cancer, disorders of the thyroid gland, inflammatory bowel disease, and migraine/headache. Among the 39 guidelines published from MICs, 13 were pregnancy-specific, and guidelines commonly provided recommendations for cardiovascular diseases [15–20], GDM [21–25], rheumatoid arthritis [26], and multiple sclerosis [21].

### Mapping of recommendations for high-priority NCD conditions

A total of 47 guidelines were identified which related to the high-priority NCD conditions identified by the WHO expert group. From these, we extracted 1834 individual recommendations.

#### Diabetes mellitus (GDM and pre-existing)

In total, 19 guidelines [21–25, 27–40] published between 2013 and 2019 provide recommendations for management of GDM (Table 2). Guidelines issued by an international or regional body were by Endocrine Society [29], WHO [30], International Federation of Gynecology and Obstetrics (FIGO) [35], and Asociación Latinoamericana de Diabetes [33] (ALAD). The remaining guidelines were from USA [27, 28, 38], UK [36, 37], Germany [32], New Zealand [39], Australia [31], and Canada [34], and six guidelines from MICs (India [21], China [23, 72], Chile [40], Georgia [25], Malaysia [24], and Thailand [22]). From the 19 guidelines, we extracted 449 recommendations, of which 299 (67%) were for screening/diagnostic or clinical interventions during antenatal care, 52 (12%) for intrapartum care, 89 (20%) for postnatal care (Additional file 6: Table 1), and eight were for health system interventions. Several guidelines covered approaches to screening for GDM (universal screening or screening high-risk women only), as well as timing and/or type of screening tests during both antenatal and postnatal care. Clinical interventions included appropriate use of pharmacological therapies, lifestyle education (particularly around nutrition and weight management), and appropriate care during labor and birth (e.g., mode and timing of delivery). More than half of guidelines provided recommendations on self-monitoring and glycemic targets during pregnancy, and indications for lactation. Health systems recommendations covered multidisciplinary care, auditing, and standards for laboratory testing. On comparing guidelines from HICs versus MICs, the guideline scope and interventions covered were broadly similar.

**Table 2:** Clinical practice guidelines for high priority conditions

No.	Author(s)	Title	Year	Country	Number of recommendations
**Diabetes mellitus**					
*Gestational diabetes*					
1	Endocrine Society [29]	Diabetes and pregnancy: an endocrine society clinical practice guideline	2013	International	32
2	The Association of Physicians of India [21]	Consensus evidence-based guidelines for management of gestational diabetes mellitus in India	2014	India	23
3	German Diabetes Association/ German Association for Gynecology and Obstetrics [32]	Gestational diabetes mellitus (GDM) diagnosis, therapy and follow-up care	2014	Germany	45
4	Ministry of Health (New Zealand) [39]	Screening, diagnosis and management of gestational diabetes in New Zealand: a clinical practice guideline	2014	New Zealand	29
5	Moyer VA, U.S. Preventive Services Task Force [38]	Screening for gestational diabetes mellitus: U.S. Preventive Services Task Force recommendation statement	2014	United States of America	3
6	World Health Organization [30]	Diagnostic criteria and classification of hyperglycaemia first detected in pregnancy: a World Health Organization Guideline	2014	International	1
7	Chinese Medical Association [23]	Diagnosis and therapy guideline of pregnancy with diabetes mellitus	2014	China	32
8	Hospital Clínico Universidad de Chile [40]	Serie guías clínicas: diabetes y embarazo (1ª parte): cuidado preconcepcional, diagnóstico y seguimiento	2015	Chile	8
9	Ministry of Labor, Health and Social Welfare [25]	Management of gestational diabetes ( )	2015	Georgia	22
10	National Institute for Health and Care Excellence [37]	Diabetes in pregnancy: management from preconception to the postnatal period	2015	United Kingdom	66
11	International Federation of Gynecology and Obstetrics [35]	The International Federation of Gynecology and Obstetrics (FIGO) Initiative on gestational diabetes mellitus: A pragmatic guide for diagnosis, management, and care	2015	International	29
12	Asociación Latinoamericana de Diabetes [33]	Guías de diagnóstico y tratamiento de la diabetes gestacional	2016	Latin America	10
13	Joint British Diabetes Societies for inpatient care [36]	Management of glycaemic control in pregnant women with diabetes on obstetric wards and delivery units	2017	United Kingdom	4
14	Ministry of Health (Malaysia) [24]	Management of Diabetes in Pregnancy	2017	Malaysia	17
15	The American College of Obstetricians and Gynecologists [28]	ACOG Practice Bulletin No. 190: Gestational Diabetes Mellitus	2017	United States of America	15
16	Royal Thai College of Obstetricians and Gynecologists [22]	Diabetes Mellitus Screening in Pregnancy	2017	Thailand	2
17	Academy of Nutrition and Dietetics [27]	Academy of Nutrition and Dietetics Gestational Diabetes Evidence-Based Nutrition Practice Guideline	2018	United States of America	18
18	Government of South Australia [31]	Gestational diabetes and diabetes mellitus	2019	Australia	19
19	Society of Obstetricians and Gynaecologists of Canada [34]	Guideline No. 393-Diabetes in Pregnancy	2019	Canada	12
*Pre-existing diabetes*					
1	National Institute for Health and Care Excellence [37]	Diabetes in pregnancy: management from preconception to the postnatal period	2015	United Kingdom	65
2	Joint British Diabetes Societies for inpatient care [36]	Management of glycaemic control in pregnant women with diabetes on obstetric wards and delivery units	2017	United Kingdom	83
3	The American College of Obstetricians and Gynecologists [41]	ACOG Practice Bulletin No. 201: Pregestational Diabetes Mellitus	2018	United States of America	9
4	Government of South Australia [31]	Gestational diabetes and diabetes mellitus	2019	Australia	126
**Chronic hypertension**					
1	Society of Obstetric Medicine of Australia and New Zealand [42]	The SOMANZ Guidelines for the Management of Hypertensive Disorders of Pregnancy 2014	2014	Australia, New Zealand	19
2	French Society of Hypertension [43]	Hypertension and pregnancy. Expert consensus statement from the French Society of Hypertension, an affiliate of the French Society of Cardiology	2016	France	15
3	Hypertension Canada [44]	Hypertension Canada's 2018 Guidelines for the Management of Hypertension in Pregnancy	2018	Canada	6
4	International Society for the Study of Hypertension in Pregnancy [45]	Hypertensive Disorders of Pregnancy: ISSHP Classification, Diagnosis, and Management Recommendations for International Practice	2018	International	28
5	Polish Society of Hypertension; Polish Cardiac Society; Polish Society of Gynecologists and Obstetricians [46]	Management of hypertension in pregnancy: prevention, diagnosis, treatment and long-term prognosis	2019	Poland	24
6	The American College of Obstetricians and Gynecologists [47]	ACOG Practice Bulletin No. 203: Chronic Hypertension in Pregnancy	2019	United States of America	11
7	Royal College of Physicians of Ireland [48]	The management of hypertension in pregnancy	2019	Ireland	21
8	Chinese Medical Association [16]	Diagnosis and treatment of hypertension and pre-eclampsia in pregnancy: a clinical practice guideline in China(2020)	2020	China	1
9	Royal Thai College of Obstetricians and Gynaecologists [20]	Management of Hypertensive Disorders in Pregnancy	2020	Thailand	9
10	Swedish Association of Obstetrics and Gynecology [49]	Riktlinjer för hypertonisjukdomar under graviditet	2021	Sweden	9
**Respiratory conditions**					
*Asthma*					
1	Government of South Australia [50]	Asthma in pregnancy	2016	Australia	79
2	National Institute for Health and Care Excellence [51]	Intrapartum care for women with existing medical conditions or obstetric complications and their babies	2019	United Kingdom	4
**Hemoglobinopathies and sickle cell disease**					
1	Royal College of Obstetricians and Gynecologists [52]	Management of Sickle Cell Disease in Pregnancy	2011	United Kingdom	136
**Mental and substance use disorders**					
*General mental disorders, bipolar disorder, psychotic disorders*					
1	beyondblue [53]	Depression and related disorders – anxiety, bipolar disorder and puerperal psychosis – in the perinatal period. A guideline for primary care health professionals	2011	Australia	6
2	Scottish Intercollegiate Guidelines Network [54]	SIGN 127: Management of perinatal mood disorders	2012	Scotland	27
3	BC Reproductive Mental Health Program [55]	Best Practice Guidelines for Mental Health Disorders in the Perinatal Period	2014	Canada	26
4	Society of Obstetricians and Gynaecologists of Canada [56]	Adolescent Pregnancy Guidelines	2015	Canada	2
5	World Federation of Societies of Biological Psychiatry [57]	Guidelines for Biological Treatment of Schizophrenia. Part 3: Update 2015 Management of special circumstances: Depression, Suicidality, substance use disorders and pregnancy and lactation	2015	International	13
6	Royal Australian and New Zealand College of Obstetricians and Gynaecologists [58]	Substance use in pregnancy	2018	Australia, New Zealand	7
*Depression and anxiety*					
1	beyondblue [53]	Depression and related disorders – anxiety, bipolar disorder and puerperal psychosis – in the perinatal period. A guideline for primary care health professionals	2011	Australia	65
2	Scottish Intercollegiate Guidelines Network [54]	SIGN 127: Management of perinatal mood disorders	2012	Scotland	23
3	Guideline Expert Advisory Committee [59]	Detection and management of mood disorders in the maternity setting: the Australian Clinical Practice Guidelines	2013	Australia	34
4	BC Reproductive Mental Health Program [55]	Best Practice Guidelines for Mental Health Disorders in the Perinatal Period	2014	Canada	66
5	World Health Organization [12]	Pregnancy, childbirth, postpartum and newborn care	2015	International	2
6	Government of South Australia [60]	Anxiety and depression in the perinatal period	2016	Australia	30
7	The American College of Obstetricians and Gynecologists [61]	Screening for Perinatal Depression	2018	United States of America	6
8	Registered Nurses’ Association of Ontario [62]	Assessment and Interventions for Perinatal Depression	2018	Canada	27
9	Royal Australian and New Zealand College of Obstetricians and Gynaecologists [58]	Substance use in pregnancy	2018	Australia, New Zealand	1
10	U.S. Preventive Services Task Force [63]	Interventions to Prevent Perinatal Depression: US Preventive Services Task Force Recommendation Statement	2019	United States of America	2
*Substance use disorder*					
1	The American College of Obstetricians and Gynecologists [64]	Committee Opinion No. 711: Opioid Use and Opioid Use Disorder in Pregnancy	2017	United States of America	10
2	Government of South Australia [65]	Substance use in pregnancy	2013	Australia	2
3	National Institute for Health and Care Excellence [66]	Antenatal and postnatal mental health: clinical management and service guidance	2014	United Kingdom	7
4	World Health Organization [67]	Guidelines for identification and management of substance use and substance use disorders in pregnancy	2014	International	17
5	Society of Obstetricians and Gynaecologists of Canada [56]	Adolescent Pregnancy Guidelines	2015	Canada	1
6	Society of Obstetricians and Gynaecologists of Canada [68]	No. 349-Substance Use in Pregnancy	2017	Canada	16
7	Royal Australian and New Zealand College of Obstetricians and Gynaecologists [58]	Substance use in pregnancy	2018	Australia, New Zealand	133
*Tobacco use*					
1	Government of South Australia [65]	Substance use in pregnancy	2013	Australia	31
2	Society of Obstetricians and Gynaecologists of Canada [68]	No. 349-Substance Use in Pregnancy	2017	Canada	2
*Alcohol use disorder*					
1	Government of South Australia [65]	Substance use in pregnancy	2013	Australia	17
2	World Health Organization [67]	Guidelines for identification and management of substance use and substance use disorders in pregnancy	2014	International	9
3	Royal Australian and New Zealand College of Obstetricians and Gynaecologists [58]	Substance use in pregnancy	2018	Australia, New Zealand	3
4	World Federation of Societies of Biological Psychiatry [69]	Guidelines for the treatment of alcohol use disorders in pregnant women	2019	International	45
5	Society of Obstetricians and Gynaecologists of Canada [70]	Screening and Counselling for Alcohol Consumption During Pregnancy	2020	Canada	26
6	British Columbia Centre on Substance Use [71]	Pregnancy Supplement – Provincial Guideline for the Clinical Management of High-Risk Drinking and Alcohol Use Disorder	2020	Canada	22

Four guidelines [31, 36, 37, 41] published between 2015 and 2019 provide recommendations for the management of pre-existing diabetes (Table 2). All guidelines were published in high-income countries by governmental or medical associations, including two issued in the UK (NICE [37] and Joint British Diabetes Societies [36]), one from USA (American College of Obstetrics and Gynecology, ACOG [41]), and one from Australia [31]. A total of 283 recommendations were identified across all guidelines. Just under half of these related to interventions during the antenatal period (45%), followed by intrapartum (34%), and the postnatal (19%) period. Only three recommendations for health system interventions were identified, covering aspects of policy and the health workforce. The scope of recommendations for screening and clinical interventions were comprehensive, including types of tests and use of repeat tests, lifestyle advice/education, pharmacological therapy, glucose monitoring and glycemic targets, birth planning, referrals, and newborn care (Additional file 6: Table 2).

#### Chronic hypertension

We identified 10 guidelines [16, 20, 42–49] published between 2014 and 2021 which provide recommendations for the management of chronic hypertension (Table 2). Only one guideline published by ACOG [47] was specifically developed on chronic hypertension management in pregnancy, while remaining guidelines focused more broadly on hypertensive disorders of pregnancy. All guidelines were issued by medical associations, predominantly from international (International Society for the Study of Hypertension in Pregnancy [45]) or high-income countries such as Australia/New Zealand [50 France [43], Canada [44], Poland [46], USA [47], Ireland [48], and Sweden [49]. Two guidelines identified were from middle-income countries—China and Thailand [16, 20]. Across the 10 guidelines, we extracted 143 recommendations, which mostly related to antenatal (70%) and postnatal care (15%), and the remaining recommendations were for the intrapartum period and health systems interventions. The scope of recommendations provided guidance for definitions and threshold values for diagnosis, appropriate use of antihypertensive therapy, and blood pressure targets (Additional file 6: Table 3). Most recommendations, particularly during intrapartum and postnatal period, were sparse and not supported by more than two guidelines. Guidelines from middle-income countries (China and Thailand) were broadly similar in scope and interventions covered compared to HICs.

#### Respiratory conditions

Two guidelines provided recommendations for asthma management (Table 2), published in Australia [50] and the UK [51]. The guideline from Australia focused on asthma only (74 recommendations), while the guideline from UK focused on intrapartum care for a range of existing medical conditions, including asthma (two recommendations). Of the 76 recommendations identified for asthma, 54 (71%) were for antenatal care, 20 (26%) for intrapartum care, and two (3%) for postnatal care (Additional file 6: Table 4). No health system recommendations were identified. The focus of recommendations varied and were predominately clinical interventions such as asthma management advice, components of routine care (e.g., monitoring by severity and control, determining frequency of appointments required, medication review, and checking inhaler technique), pharmacological therapy, and management of special situation such as acute exacerbations, or poorly controlled asthma. No guidelines were identified for the management of chronic obstructive pulmonary disease.

#### Hemoglobinopathies and sickle cell disease

One guideline published in 2011 by Royal College of Obstetricians and Gynecologists [52] provided 136 recommendations for the management of sickle cell disease in pregnancy (Table 2, Additional file 6: Table 5). A comprehensive range of recommendations were provided for screening (type of assessments and tests), clinical care (e.g., components of routine care, pharmacological therapy, acute pain management, birth planning), and health system interventions (e.g., use of clinical protocols, health workforce, referral pathways). No guidelines were identified for the management hemoglobinopathies.

#### Mental and substance use disorders

A total of 20 guidelines were identified for mental and substance use disorders, which provided recommendations for general mental disorders, bipolar disorder or psychotic disorders [53–58], depression and anxiety [53–55, 58–63, 67], substance use disorders [56, 58, 64–68], alcohol use disorders [58, 65, 69–71], and tobacco use [65, 67, 68] (Table 2, Additional file 6: Tables 6-10). All guidelines were developed by international bodies [30, 57, 69] or from HICs (Australia and/or New Zealand [53, 58–60, 65], Canada [55, 56, 62, 68], USA [61, 63, 64], and Scotland [54]).

In total, 81 recommendations pertained to general mood disorders, bipolar disorder, psychotic disorder, schizophrenia, and suicide and infanticide risk (Additional file 6: Table 6). Recommendations for screening during antenatal and postnatal period included assessment by personal or family history, and the use of Edinburgh Postnatal Depression Scale (EPDS) for pregnant adolescents. Clinical interventions covered the development of an individualized treatment plan, pharmacological treatment, and referrals to psychiatric services. At the health system level, involvement of a multidisciplinary care team (psychiatrists, gynecologists, pediatricians, and midwives) were recommended. One guideline specified the need for a nationally managed clinical network for perinatal mental health which should coordinate provision of services, establish pathways for referral and management, and establish competencies and training resources for health professionals. No recommendations were identified relating specifically to the intrapartum period.

For depression and anxiety, 256 recommendations covered psychosocial assessment using tools such as EPDS, pharmacotherapy (risk-benefit analysis, medication review, and dosage adjustment), and psychotherapy (e.g., cognitive behavioral therapy, interpersonal psychotherapy) (Additional file 6: Table 7). Recommendations for women at high risk—such as suicidal or severely depressed—included involvement of partner support, and appropriate use of home treatment or hospitalization. Further clinical assessment were also recommended, including referral to psychiatric services, and actively supporting women to use mental health services and counselling interventions. During the postnatal period, recommendations for breastfeeding included indications and possible risks associated with pharmacotherapy. Additionally, recommendations covered neonatal monitoring for adverse effects associated with maternal use of psychoactive therapeutic agents. At the health system level, recommendations focused on availability of local guidelines, establishing health provider competencies, ensuring service availability for diagnosis and treatment, and providing education and training for involved health professionals.

The breadth of interventions for substance use disorders varied by the type of substance (Additional file 6: Table 8). A total 186 recommendations covered interventions for screening and routine assessment, as well as specific recommendations for the management of women using amphetamines, benzodiazepines, cannabis, opioids, and psychostimulants. Compared to other high-priority conditions, health systems made up a relatively higher proportion of recommendations (12%) and covered the roles of case managers in coordinating care between multiple services (drug and alcohol services, family support services, general practitioners, probation and parole services, and community welfare organizations), statutory reporting, liaising with child protection agencies, and ensuring the availability of services for treatment and relapse protection programs.

For tobacco use, two guidelines were identified which provided 33 recommendations (Additional file 6: Table 9). For alcohol use disorders, six guidelines were identified which provided 122 recommendations (Additional file 6: Table 10). Recommendations mainly covered screening and clinical interventions during the antenatal and postnatal periods. For tobacco use, recommendations included universal assessment of smoking status, maternal education on the harmful effects of smoking and advice for smoking cessation, engaging with partners for support, providing psychosocial and/or pharmacotherapy interventions, and strategies for relapse prevention and management. For alcohol use disorders, screening using validated questionnaires such as the Alcohol Use Disorders Identification Test was recommended, as well as interventions to provide advice on abstinence, managing alcohol withdrawal syndrome, indications for breastfeeding, and conducting assessments and monitoring for fetal alcohol spectrum disorder. For both conditions, health system recommendations included ensuring availability and access for prevention and management services across existing sexual, reproductive, and childcare, or by community-based interventions. No clinical interventions were recommended during the intrapartum period.

## Discussion

### Main findings

We found that across 405 identified guidelines, recommendations provided were largely focused on a small number NCD conditions with higher prevalence in pregnant women such as GDM and mental health disorders. Other clinically important conditions were found to have limited or no guidelines, demonstrated by our guideline mapping of which less than one-third of all guidelines accounted for nine of 14 NCD categories (neurological conditions, digestive diseases, genitourinary disease, congenital anomalies, musculoskeletal disease, respiratory diseases, skin diseases, oral conditions, and sense organ diseases). This distribution might reflect stronger international collaborative efforts to address higher-profile conditions which were specified in the United Nation’s Sustainable Development Goals such as cancers, cardiovascular diseases, chronic respiratory diseases, and pre-existing diabetes mellitus [73]. Such international goals shape allocation of resources, but may mean that other, less prevalent, conditions are overlooked. There is a need to ensure that up-to-date guidelines are available to promote evidence-based care for all NCD conditions, regardless of prevalence. To our knowledge, this is the first scoping review of published guidelines for a comprehensive range of NCDs in the maternity care context. Findings of this study has proved critical for WHO in planning their future guideline development activities for high-priority conditions (diabetes, chronic hypertension, respiratory conditions, hemoglobinopathies and sickle cell disease, and mental and substance use disorders).

Only one guideline, published by NICE [51], addressed a range of different NCD conditions as the main topic. All other guidelines focused on a single disease or condition, highlighting the current “vertical” approach to NCDs in pregnant or postpartum women. However, this approach is not well-suited to address the issue of multi-morbidity affecting pregnant women, where a more integrated, holistic approach to clinical care is required. Many NCD conditions co-occur due to shared risk factors. Additionally, having one NCD condition can predispose women to develop another condition. Evidence from the UK found that more than 40% of pregnant women had multi-morbidities and 70% of women with multi-morbidities had a mental health condition [74]. These complex relationships between NCDs and the lack of integrated NCD guidelines identified highlight the need to transition away from the “single disease” approach in current guidelines, towards a more woman-centered approach which facilitates the integration of a range of NCD care recommendations with maternity service provision.

One of the major challenges for NCD management is ensuring delivery of effective care in limited-resource settings. An estimated 295,000 maternal deaths occur worldwide each year, 99% of which occur in low- and middle-income countries (LMICs), with 14.8% due to pre-existing medical conditions [75]. Most guidelines were published in HICs, with less than 10% of the guidelines found published in MICs and no guidelines identified in low-income countries. Notably, two countries (India and South Africa) had published a number of NCD-related guidelines for national use, and both have a considerable burden of NCD-related maternal morbidity and mortality [76, 77]. This finding highlights the need for guidelines tailored to limited-resource contexts, particularly those with a high burden of NCD-related morbidity and mortality. By strengthening NCD prevention and management, it will contribute as a critical component to reach SDG targets for reduction of both maternal mortality, and premature mortality due to NCDs [73]. The difference in guideline availability between HICs and LMICs is consistent with previous studies which compared NCD guidelines for non-pregnant adults [78–81]. This could be partly explained by low prioritization of NCD-related activities in national health policies, or limited human and financial resources to support guideline development. Thus, efforts to develop and implement context-appropriate guidelines are urgently required to help improve NCD-related care in LMICs.

This mapping of over 1800 recommendations from 47 guidelines on high-priority conditions revealed that most recommendations pertained to antenatal interventions, such as early detection, screening tools, and pharmacological treatment for diseases and their complications. Our generated map of interventions was used to guide development of future WHO maternal health guidelines, as well as identified gaps in currently recommended practice. While the antenatal period is longer and presents more opportunities for clinical intervention, the intrapartum and postnatal periods remain important for provision of NCD care to reduce the risk of obstetric and perinatal complications [82]. Additionally, the postnatal period is a critical window for optimizing NCD prevention and management in long-term, as well as ensuring continuity into primary or specialist care [83]. Given the variation in quality and depth of recommendations, it was challenging to identify which group of interventions would be considered part of an essential “minimum package” for managing NCDs in pregnant and postpartum women. Future guideline development efforts could consider focusing on the most critical interventions for reducing NCD-related maternal morbidity and mortality, as well as interventions to reduce variation in quality of NCD-related care. Future analyses that compare recommended interventions from different countries might help explain national differences in coverage or quality in NCD-related maternity care, or NCD-related health outcomes.

While guidelines are important and useful tools, the translation of knowledge into improved health outcomes is largely dependent on guideline uptake. Limited implementation of guidelines in maternity care contexts are frequently reported in LMICs [84, 85], due to key barriers such as low awareness of the guideline and/or disease burden, physician disagreement or perceived lack of relevance, and inadequate training or infrastructure to promote intervention use [86]. Thus, in development of future NCD guidelines, policymakers should consider the contextual factors in low-resource settings which may affect the adoption and uptake of recommendations. This should be supplemented with ongoing research to explore barriers and facilitators of guideline implementation in antenatal, intrapartum, and postnatal care particularly in LMICs.

To assess as many guidelines as possible, we undertook a broad, comprehensive search in four databases and searched websites of over 160 national and international societies, colleges, and organizations. This study also focused on selected high-priority conditions informed by judgements of expert stakeholders participating in a WHO technical consultation. We also adhered to best-practice principles in conducting the searches, duplicate screening, and data extraction in this scoping review. However, we recognize that certain limitations exist. While we aimed to capture as many guidelines as possible, guidelines are not well-indexed in electronic databases, and it is possible some eligible guidelines may not have been identified. Other evidence-based products are used to support clinical decision-making but were not included in this review, such as hospital protocols, job aids or textbooks. As a scoping review, we did not undertake quality appraisal of individual guidelines, though to partially mitigate this the eligibility criteria specified including only those guidelines where recommendations were based on a systematic review of evidence. Detailed quality assessments of individual guidelines using validated tools such as AGREE II [87] could help distinguish higher-quality guidelines. We also acknowledge that most identified guidelines were from HICs, and it is not known whether these guidelines are applicable or appropriate for limited-resource settings. In addition, more detailed comparisons at the level of individual recommendations for the same condition would help identify where and why different guidelines disagree. Lastly, though this review was reasonably broad, an expanded focus to explore guidelines on pre-conception care (particularly regarding diet and nutrition) would provide value to ensuring continuity of care. Similarly, as obesity-related guidelines for maternity care settings were not included in this study, future exploration in this area is warranted.

## Conclusions

This scoping review identified 405 guidelines on the management of NCDs during antenatal, intrapartum, and postnatal care. Overall, this study provides a robust assessment of the availability, focus, and scope of current available guidelines on NCD management within maternal health services, which will inform the scope of WHO’s future guideline development activities. There is a clear need for NCD-related maternity guidelines that are developed specifically for limited-resource settings.

## Supplementary Information

**Additional file 1: Table 1.** PRISMA scoping review reporting checklist.

**Additional file 2: Table 1.** Non-communicable disease categories for analysis and selected high-priority conditions.

**Additional file 3. **Search strategy. **Table 1.** Search strategy for PubMed. **Table 2.** Search strategy for Global Index Medicus. **Table 3.** Search strategy for TRIP database.

**Additional file 4: Table 1.** Websites of organizations, societies, associations, and colleges identified for searching of relevant guidelines.

**Additional file 5: Table 1.** Characteristics of identified guidelines for the management of non-communicable diseases during antenatal, intrapartum, and postnatal care by disease category.

**Additional file 6. **Scope of recommendations for high priority conditions. **Table 1.** Gestational diabetes mellitus (GDM). **Table 2.** Diabetes mellitus (pre-existing). **Table 3.** Chronic hypertension. **Table 4.** Asthma. **Table 5.** Sickle cell disorder. **Table 6.** General mental disorders, bipolar disorder, psychotic disorders. **Table 7.** Depression and anxiety. **Table 8.** Substance use disorders. **Table 9.** Tobacco use. **Table 10.** Alcohol use disorder.

## Data Availability

All data generated or analyzed during this study are included in this published article and its Additional files.

## References

[1] Barnes SB, Ramanarayanan D, Amin N. The unseen side of pregnancy: non-communicable diseases and maternal health. https://www.wilsoncenter.org/sites/default/files/media/uploads/documents/TheUnseenSideofPregnancy.pdf. Accessed 01 May 2022.

[2] NCD Alliance. Noncommunicable diseases: a priority for women’s health and development. https://ncdalliance.org/sites/default/files/resource_files/Non%20Communicable%20Diseases%20A%20priority%20for%20womens%27s%20health%20and%20development.pdf. Accessed 01 May 2022.

[3] Kapur A, Hod M (2020). Maternal health and non-communicable disease prevention: an investment case for the post COVID-19 world and need for better health economic data. Int J Gynaecol Obstet.

[4] Ramlakhan KP, Johnson MR, Roos-Hesselink JW (2020). Pregnancy and cardiovascular disease. Nat Rev Cardiol.

[5] Institute of Medicine Committee on Standards for Developing Trustworthy Clinical Practice G (2011). Clinical Practice Guidelines We Can Trust.

[6] Mussa J, Meltzer S, Bond R, Garfield N, Dasgupta K (2021). Trends in national Canadian guideline recommendations for the screening and diagnosis of gestational diabetes mellitus over the years: a scoping review. Int J Environ Res Public Health.

[7] Al Khaja KA, Sequeira RP, Alkhaja AK, Damanhori AH (2014). Drug treatment of hypertension in pregnancy: a critical review of adult guideline recommendations. J Hypertens.

[8] Khan I, Okosieme O, Lazarus J (2017). Antithyroid drug therapy in pregnancy: a review of guideline recommendations. Expert Rev Endocrinol Metab.

[9] Moher D, Shamseer L, Clarke M, Ghersi D, Liberati A, Petticrew M (2015). Preferred reporting items for systematic review and meta-analysis protocols (PRISMA-P) 2015 statement. Syst Rev.

[10] World Health Organization. International classification of health interventions (ICHI). https://www.who.int/standards/classifications/international-classification-of-health-interventions. Accessed 01 May 2022.

[11] World Health Organization. WHO methods and data sources for country-level causes of death 2000-2019. https://www.who.int/docs/default-source/gho-documents/global-health-estimates/ghe2019_cod_methods.pdf?sfvrsn=37bcfacc_5. Accessed 01 May 2022.

[12] World Health Organization, United Nations Population Fund, World Bank, United Nations Children's Fund (UNICEF). Pregnancy, childbirth, postpartum and newborn care: A guide for essential practice (3rd edition). https://www.who.int/publications/i/item/9789241549356. Accessed 01 May 2022. 26561684

[13] Canada's Drug and Health Technology Agency (CADTH). Strings attached: CADTH database search filters. https://www.cadth.ca/strings-attached-cadths-database-search-filters. Accessed 22 Dec 2021.

[14] Veritas Health Innovation. Covidence systematic review software. www.covidence.org. Accessed 01 Jul 2021.

[15] Ministry of Labor Health and Social Welfare. Cardiovascular disease management during pregnancy. http://www.goga.org.ge/img/%E1%83%99%E1%83%90%E1%83%A0%E1%83%93%E1%83%98%E1%83%9D%E1%83%95%E1%83%90%E1%83%A1%E1%83%99%E1%83%A3%E1%83%9A%E1%83%A3%E1%83%A0%E1%83%92%E1%83%90%E1%83%98%E1%83%93%E1%83%9A%E1%83%90%E1%83%98%E1%83%9C%E1%83%98.pdf. Accessed 01 May 2022.

[16] Hypertensive Disorders in Pregnancy Subgroup - Chinese Society of Obstetrics and Gynecology/Chinese Medical Association (2020). Diagnosis and treatment of hypertension and pre-eclampsia in pregnancy: a clinical practice guideline in China. Zhonghua Fu Chan Ke Za Zhi.

[17] Obstetrics Subgroup - Chinese Society of Obstetrics and Gynocology/Chinese Medical Association (2016). Expert consensus document of the diagnosis and treatment of pregnancy with heart disease. Zhonghua Fu Chan Ke Za Zhi.

[18] Ministry of Health Malaysia. Heart Disease in Pregnancy. https://www.malaysianheart.org/files/583aacd86e27d.pdf. Accessed 01 May 2022.

[19] Moodley J, Soma-Pillay P, Buchmann E, Pattinson RC (2019). Hypertensive disorders in pregnancy: 2019 National guideline. S Afr Med J.

[20] The Royal Thai College of Obstetricians and Gynaecologists. Managment of Hypertensive Disorders in Pregnancy. http://www.rtcog.or.th/home/wp-content/uploads/2020/10/OB-63-021_Management-of-Hypertensive-Disorders-in-Pregnancy07Oct20.pdf. Accessed 01 May 2022.

[21] Seshiah V, Banerjee S, Balaji V, Muruganathan A, Das AK (2014). Consensus evidence-based guidelines for management of gestational diabetes mellitus in India. J Assoc Physicians India.

[22] The Royal Thai College of Obstetricians and Gynaecologists. Diabetes Mellitus Screening in Pregnancy. http://www.rtcog.or.th/home/wp-content/uploads/2017/05/OB-010_Diabetes-meltitus-screeing-in-pregnancy.pdf. Accessed 01 May 2022.

[23] Obstetrics Subgroup - Chinese Society of Obstetrics and Gynecology/Chinese Medical Association, Group of Pregnancy with Diabetes Mellitus - Chinese Society of Perinatal Medicine/Chinese Medical Association (2014). Diagnosis and therapy guideline of pregnancy with diabetes mellitus. Zhonghua Fu Chan Ke Za Zhi.

[24] Ministry of Health Malaysia. Management of Diabetes in Pregnancy. https://www.moh.gov.my/moh/resources/Penerbitan/CPG/Endocrine/1a.pdf. Accessed 01 May 2022.

[25] Ministry of Labor Health and Social Welfare. Management of gestational diabetes. https://www.moh.gov.ge/uploads/guidelines/2017/06/01/88ccc5e850b980e984bd653c3b854778.pdf. Accessed 01 May 2022.

[26] Saavedra Salinas MÁ, Barrera Cruz A, Cabral Castañeda AR, Jara Quezada LJ, Arce-Salinas CA, Álvarez Nemegyei J (2015). Clinical practice guidelines for the management of pregnancy in women with autoimmune rheumatic diseases of the Mexican College of Rheumatology. Part II. Reumatol Clin.

[27] Duarte-Gardea MO, Gonzales-Pacheco DM, Reader DM, Thomas AM, Wang SR, Gregory RP (2018). Academy of Nutrition and Dietetics Gestational Diabetes Evidence-Based Nutrition Practice Guideline. J Acad Nutr Diet.

[28] The American College of Obstetricians and Gynecologists (2018). ACOG Practice Bulletin No. 190: Gestational Diabetes Mellitus. Obstet Gynecol.

[29] Blumer I, Hadar E, Hadden DR, Jovanovič L, Mestman JH, Murad MH (2013). Diabetes and pregnancy: an endocrine society clinical practice guideline. J Clin Endocrinol Metab.

[30] World Health Organization. Diagnostic criteria and classification of hyperglycaemia first detected in pregnancy. https://apps.who.int/iris/handle/10665/85975. Accessed 01 Jul 2021. 24199271

[31] Government of South Australia. Gestational diabetes and diabetes mellitus. https://www.sahealth.sa.gov.au/wps/wcm/connect/146238004ee2144cb404bdd150ce4f37/Diabetes+Mellitus+and+Gestational+Diabetes_July2015.pdf?MOD=AJPERES&CACHEID=ROOTWORKSPACE-146238004ee2144cb404bdd150ce4f37-mhbYUex. Accessed 01 Jul 2021.

[32] Kleinwechter H, Schäfer-Graf U, Bührer C, Hoesli I, Kainer F, Kautzky-Willer A (2014). Gestational diabetes mellitus (GDM) diagnosis, therapy and follow-up care: Practice Guideline of the German Diabetes Association(DDG) and the German Association for Gynaecologyand Obstetrics (DGGG). Exp Clin Endocrinol Diabetes.

[33] Asociación Latinoamericana de Diabetes. Guías de diagnóstico y tratamiento de la diabetes gestacional. https://www.entrerios.gov.ar/msalud/wp-content/uploads/2013/05/publicacion-guias-dg-alad_v6_n4_155-169-4.pdf. Accessed 01 Jul 2021.

[34] Berger H, Gagnon R, Sermer M (2019). Guideline No. 393-Diabetes in Pregnancy. J Obstet Gynaecol Can.

[35] Hod M, Kapur A, Sacks DA, Hadar E, Agarwal M, Di Renzo GC (2015). The International Federation of Gynecology and Obstetrics (FIGO) Initiative on gestational diabetes mellitus: a pragmatic guide for diagnosis, management, and care. Int J Gynaecol Obstet.

[36] Joint British Diabetes Societies for Inpatient Care (JBDS–IP). Management of glycaemic control in pregnant women with diabetes on obstetric wards and delivery units. https://www.diabetes.org.uk/resources-s3/2017-10/JBDS%20Pregnancy%202017%2020.10.17_0.pdf. Accessed 01 Jul 2021.

[37] National Collaborating Centre for Women's Children's Health. In: National Institute for Health and Care Excellence: clinical guidelines. Diabetes in Pregnancy: Management of Diabetes and Its Complications from Preconception to the Postnatal Period. London: National Institute for Health and Care Excellence (UK); 2015. 25950069

[38] Moyer VA (2014). Screening for gestational diabetes mellitus: U.S. Preventive Services Task Force recommendation statement. Ann Intern Med.

[39] Ministry of Health (New Zealand). Screening, diagnosis and management of gestational diabetes in New Zealand: a clinical practice guideline. https://www.health.govt.nz/system/files/documents/publications/screening-diagnosis-management-of-gestational-diabetes-in-nz-clinical-practive-guideline-dec14-v2.pdf. Accessed 01 Jul 2021.

[40] Hospital Clínico Universidad de Chile (2015). Serie guías clínicas: diabetes y embarazo (1ª parte): cuidado preconcepcional, diagnóstico y seguimiento. Rev Hosp Clín Univ Chile.

[41] American College of Gynecologists and Obstetricians (2018). ACOG Practice Bulletin No. 201: Pregestational Diabetes Mellitus. Obstet Gynecol.

[42] Lowe SA, Bowyer L, Lust K, McMahon LP, Morton M, North RA (2015). SOMANZ guidelines for the management of hypertensive disorders of pregnancy 2014. Aust N Z J Obstet Gynaecol.

[43] Mounier-Vehier C, Amar J, Boivin JM, Denolle T, Fauvel JP, Plu-Bureau G (2016). Hypertension and pregnancy. Expert consensus statement from the French Society of Hypertension, an affiliate of the French Society of Cardiology. Presse Med.

[44] Butalia S, Audibert F, Côté AM, Firoz T, Logan AG, Magee LA (2018). Hypertension Canada’s 2018 Guidelines for the Management of Hypertension in Pregnancy. Can J Cardiol.

[45] Brown MA, Magee LA, Kenny LC, Karumanchi SA, McCarthy FP, Saito S (2018). Hypertensive disorders of pregnancy: ISSHP classification, diagnosis, and management recommendations for international practice. Hypertension..

[46] Prejbisz A, Dobrowolski P, Kosiński P, Bomba-Opoń D, Adamczak M, Bekiesińska-Figatowska M (2019). Management of hypertension in pregnancy: prevention, diagnosis, treatment and long-term prognosis. Kardiol Pol.

[47] American College of Obstetrics and Gynecologists (2019). ACOG Practice Bulletin No. 203: Chronic Hypertension in Pregnancy. Obstet Gynecol.

[48] Royal College of Physicians of Ireland (RCPI). The management of hypertension in pregnancy. https://rcpi-live-cdn.s3.amazonaws.com/wp-content/uploads/2017/02/Hypertension-Guideline_approved_120716-1.pdf. Accessed 01 Jul 2021.

[49] Swedish Society of Obstetrics and Gynecology. Riktlinjer för hypertonisjukdomar under graviditet. https://www.sfog.se/media/337263/hypertonisjukdomar-under-graviditet-sfog-2019-10-23-reviderad-210121.pdf. Accessed 01 Jul 2021.

[50] Government of South Australia. Asthma in Pregnancy. https://www.sahealth.sa.gov.au/wps/wcm/connect/ea28ed004ee1da8eacbeadd150ce4f37/Asthma-pregnancy-WCHN-PPG-20052012.pdf?MOD=AJPERES&CACHEID=ROOTWORKSPACE-ea28ed004ee1da8eacbeadd150ce4f37-nxzd1bA. Accessed 01 Jul 2021.

[51] National Guideline Alliance (2019). National Institute for Health and Care Excellence: Clinical Guidelines. Intrapartum care for women with existing medical conditions or obstetric complications and their babies.

[52] Royal College of Obstetricians and Gynaecologists. Management of Sickle Cell Disease in Pregnancy. https://www.rcog.org.uk/globalassets/documents/guidelines/gtg_61.pdf. Accessed 01 Jul 2021.

[53] beyondblue. Depression and related disorders – anxiety, bipolar disorder and puerperal psychosis – in the perinatal period. A guideline for primary care health professionals. https://cope.org.au/wp-content/uploads/2013/12/Perinatal-Mental-Health-Clinical-Practice-Guidelines.pdf. Accessed 01 Jul 2021.

[54] Scottish Intercollegiate Guidelines Network (SIGN). Management of perinatal mood disorders. https://www.sign.ac.uk/assets/sign127_update.pdf. Accessed 01 Jul 2021.

[55] BC Reproductive Mental Health Program. Best Practice Guidelines for Mental Health Disorders in the Perinatal Period. http://www.perinatalservicesbc.ca/Documents/Guidelines-Standards/Maternal/MentalHealthDisordersGuideline.pdf. Accessed 01 Jul 2021.

[56] Fleming N, O'Driscoll T, Becker G, Spitzer RF (2015). Adolescent Pregnancy Guidelines. J Obstet Gynaecol Can.

[57] Hasan A, Falkai P, Wobrock T, Lieberman J, Glenthøj B, Gattaz WF (2015). World Federation of Societies of Biological Psychiatry (WFSBP) Guidelines for Biological Treatment of Schizophrenia. Part 3: Update 2015 Management of special circumstances: Depression, Suicidality, substance use disorders and pregnancy and lactation. World J Biol Psychiatry.

[58] The Royal Australian and New Zealand College of Obstetricians and Gynaecologists. Substance use in pregnancy. https://ranzcog.edu.au/RANZCOG_SITE/media/RANZCOG-MEDIA/Women%27s%20Health/Statement%20and%20guidelines/Clinical-Obstetrics/Substance-use-in-pregnancy-(C-Obs-55)-March-2018.pdf?ext=.pdf. Accessed 01 Jul 2021.

[59] Austin MP, Middleton P, Reilly NM, Highet NJ (2013). Detection and management of mood disorders in the maternity setting: the Australian Clinical Practice Guidelines. Women Birth.

[60] Government of South Australia. Anxiety and depression in the perinatal period. https://www.sahealth.sa.gov.au/wps/wcm/connect/c7c0ccf9-b704-4411-9b47-fbf777ac0829/Anxiety+and+Depression+in+the+Perinatal+Period_PPG_v1_0.pdf?MOD=AJPERES&CACHEID=ROOTWORKSPACE-c7c0ccf9-b704-4411-9b47-fbf777ac0829-nGzKLbc. Accessed 01 Jul 2021.

[61] American College of Obstetricians and Gynecologists. ACOG Committee Opinion No. 757: Screening for Perinatal Depression. Obstet Gynecol. 2018;132(5):e208–12. 10.1097/AOG.0000000000002927 30629567

[62] Registered Nurses’ Association of Ontario. Assessment and Interventions for Perinatal Depression. https://rnao.ca/bpg/guidelines/assessment-and-interventions-perinatal-depression. Accessed 01 Jul 2021.

[63] Curry SJ, Krist AH, Owens DK, Barry MJ, Caughey AB, Davidson KW (2019). Interventions to Prevent Perinatal Depression: US Preventive Services Task Force Recommendation Statement. JAMA..

[64] American College of Obstetricians and Gynecologists (2017). Committee Opinion No. 711: Opioid Use and Opioid Use Disorder in Pregnancy. Obstet Gynecol.

[65] Government of South Australia. Substance use in Pregnancy. https://www.sahealth.sa.gov.au/wps/wcm/connect/fad90e004eede261b572b76a7ac0d6e4/Substance+use+in+pregnancy_May2014.pdf?MOD=AJPERES&CACHEID=ROOTWORKSPACE-fad90e004eede261b572b76a7ac0d6e4-n5j0wVl. Accessed 01 Jul 2021.

[66] National Institute for Health and Care Excellence (NICE) (2014). Antenatal and postnatal mental health: clinical management and service guidance.

[67] World Health Organization. Guidelines for identification and management of substance use and substance use disorders in pregnancy. https://www.who.int/publications/i/item/9789241548731. Accessed 01 Jul 2021. 24783312

[68] Ordean A, Wong S, Graves L (2017). No. 349-Substance Use in Pregnancy. J Obstet Gynaecol Can.

[69] Thibaut F, Chagraoui A, Buckley L, Gressier F, Labad J, Lamy S (2019). WFSBP and IAWMH Guidelines for the treatment of alcohol use disorders in pregnant women. World J Biol Psychiatry.

[70] Graves L, Carson G, Poole N, Patel T, Bigalky J, Green CR (2020). Guideline No. 405: Screening and counselling for alcohol consumption during pregnancy. J Obstet Gynaecol Can.

[71] British Columbia Centre on Substance Use (BCCSU). Pregnancy Supplement – Provincial Guideline for the Clinical Management of High-Risk Drinking and Alcohol Use Disorder. https://www.bccsu.ca/wp-content/uploads/2021/03/Pregnancy-Supplement-Provincial-Guideline-for-the-Clinical-Management-of-High-Risk-Drinking-and-Alcohol-Use-Disorder.pdf. Accessed 01 Jul 2021.

[72] GBD SDG Collaborators (2016). Measuring the health-related Sustainable Development Goals in 188 countries: a baseline analysis from the Global Burden of Disease Study 2015. Lancet..

[73] United Nations. Transforming our world: the 2030 agenda for sustainable development. https://sdgs.un.org/2030agenda. Accessed 08 Dec 2021.

[74] Lee SI, Azcoaga-Lorenzo A, Agrawal U, Kennedy JI, Fagbamigbe AF, Hope H (2021). Epidemiology of pre-existing multimorbidity in pregnant women in the UK in 2018: a cross-sectional study. Lancet..

[75] Say L, Chou D, Gemmill A, Tunçalp Ö, Moller AB, Daniels J (2014). Global causes of maternal death: a WHO systematic analysis. Lancet Glob Health.

[76] The George Institute for Global Health India. High-risk pregnancy and non-communicable diseases in India. https://www.georgeinstitute.org.in/sites/default/files/smarthealth_pregnancy_workshops.pdf. Accessed 01 Jul 2021.

[77] Yaya S, Reddy KS, Belizán JM, Pingray V (2020). Non-communicable diseases and reproductive health in sub-Saharan Africa: bridging the policy-implementation gaps. Reprod Health.

[78] Tabyshova A, Hurst JR, Soriano JB, Checkley W, Wan-Chun Huang E, Trofor AC (2021). Gaps in COPD guidelines of low- and middle-income countries: a systematic scoping review. Chest..

[79] Owolabi M, Olowoyo P, Miranda JJ, Akinyemi R, Feng W, Yaria J (2016). Gaps in hypertension guidelines in low- and middle-income versus high-income countries: a systematic review. Hypertension..

[80] Bayona H, Owolabi M, Feng W, Olowoyo P, Yaria J, Akinyemi R (2017). A systematic comparison of key features of ischemic stroke prevention guidelines in low- and middle-income vs. high-income countries. J Neurol Sci.

[81] Owolabi MO, Yaria JO, Daivadanam M, Makanjuola AI, Parker G, Oldenburg B (2018). Gaps in guidelines for the management of diabetes in low- and middle-income versus high-income countries-a systematic review. Diabetes Care.

[82] Admon LK, Winkelman TNA, Heisler M, Dalton VK (2018). Obstetric outcomes and delivery-related health care utilization and costs among pregnant women with multiple chronic conditions. Prev Chronic Dis.

[83] Miller S, Abalos E, Chamillard M, Ciapponi A, Colaci D, Comandé D (2016). Beyond too little, too late and too much, too soon: a pathway towards evidence-based, respectful maternity care worldwide. Lancet..

[84] Seyoum T, Alemayehu M, Christensson K, Lindgren H (2020). Client factors affect provider adherence to guidelines during first antenatal care in public health facilities, Ethiopia: a multi-center cross-sectional study. Ethiop J Health Sci.

[85] Escobar CM, Grünebaum A, Nam EY, Olson AT, Anzai Y, Benedetto-Anzai MT (2020). Non-adherence to labor guidelines in cesarean sections done for failed induction and arrest of dilation. J Perinat Med.

[86] Millington S, Arstall M, Dekker G, Magarey J, Clark R (2020). Adherence to clinical practice guidelines for South Australian pregnant women with cardiac conditions between 2003 and 2013. PLoS One.

[87] Brouwers MC, Kho ME, Browman GP, Burgers JS, Cluzeau F, Feder G (2010). AGREE II: advancing guideline development, reporting and evaluation in health care. CMAJ..

