# Supplementary material for: Prevention and control of non-communicable diseases in antenatal, intrapartum, and postnatal care: a systematic scoping review of clinical practice guidelines since 2011
Source: BMC Med. 2022 Sep 20;20:305. doi: 10.1186/s12916-022-02508-9 (PMC9487084; doi:10.1186/s12916-022-02508-9)
Supplement: Supplementary file 2 — Additional file 2: Table 1. Non-communicable disease categories for analysis and selected high-priority conditions. [file 12916_2022_2508_MOESM2_ESM.docx]

**Additional file 2. Prevention and control of non-communicable diseases in antenatal, intrapartum, and postpartum care: a systematic scoping review of clinical practice guidelines since 2011**

**Table 1. Non-communicable disease categories for analysis and selected high-priority conditions**

| Non-communicable disease categories | Included conditions^†^ |
| --- | --- |
| Malignant neoplasms | - All cancers and other neoplasms |
| Diabetes mellitus | - **Diabetes mellitus** - **Gestational diabetes mellitus** |
| Endocrine, blood, immune disorders | - **Thalassemia** - **Sickle cell disorders and trait** - **Other haemoglobinopathies and hemolytic anemias** |
| Mental and substance use disorders | - **Depressive disorders** - **Bipolar disorder** - Schizophrenia - **Alcohol use disorders** - **Drug use disorders** - **Anxiety disorders** - Eating disorders - Autism and Asperger syndrome |
| Neurological conditions | - Alzheimer disease and other dementias - Parkinson disease - Epilepsy - Multiple sclerosis |
| Sense organ diseases | - Glaucoma - Cataracts - Uncorrected refractive errors - Macular degeneration |
| Cardiovascular diseases | - Rheumatic heart disease - Hypertensive heart disease (including **chronic hypertension**) - Ischemic heart disease - Stroke - Cardiomyopathy, myocarditis, endocarditis |
| Respiratory diseases | - **Chronic obstructive pulmonary disease** - **Asthma** |
| Digestive diseases | - Peptic ulcer disease - Cirrhosis of the liver - Appendicitis - Gastritis and duodenitis - Paralytic ileus and intestinal obstruction - Inflammatory bowel disease - Gallbladder and biliary diseases |
| Genitourinary diseases | - Kidney diseases - Gynecological diseases |
| Skin diseases | Any skin diseases (ICD-10 codes L00-L98) |
| Musculoskeletal diseases | - Rheumatoid arthritis - Osteoarthritis - Gout - Back and neck pain |
| Congenital anomalies | Any congenital anomalies, including neural tube defects, cleft lip and cleft palate, down syndrome, congenital heart anomalies |
| Oral conditions | Any oral conditions, including dental caries, periodontal disease, edentulism |

^*^ High-priority conditions identified for WHO guideline development are in bold.
^†^ Categories and conditions adapted from World Health Organization Global Health Estimates. Listed included conditions are not exhaustive. A full list of conditions and their ICD codes are available from WHO methods and data sources for country-level causes of death 2000-2019 (Global Health Estimates Technical Paper WHO/DDI/DNA/GHE/2020.2, available from: <https://www.who.int/docs/default-source/gho-documents/global-health-estimates/ghe2019_cod_methods.pdf?sfvrsn=37bcfacc_5>. Accessed 02 May 2022)
