# Supplementary material for: Prevention and control of non-communicable diseases in antenatal, intrapartum, and postnatal care: a systematic scoping review of clinical practice guidelines since 2011
Source: BMC Med. 2022 Sep 20;20:305. doi: 10.1186/s12916-022-02508-9 (PMC9487084; doi:10.1186/s12916-022-02508-9)
Supplement: Supplementary file 3 — Additional file 3. Search strategy. Table 1. Search strategy for PubMed. Table 2. Search strategy for Global Index Medicus. Table 3. Search strategy for TRIP database. [file 12916_2022_2508_MOESM3_ESM.docx]

**Additional file 3. Prevention and control of non-communicable diseases in antenatal, intrapartum, and postpartum care: a systematic scoping review of clinical practice guidelines since 2011**

**Table 1. Search strategy for PubMed**

| **No.** | **Query** |
| --- | --- |
| 1 | Pregnant Women[MeSH] OR Pregnant[MeSH] OR Postpartum Period[MeSH] OR Prenatal Care[MeSH] OR Perinatal Care[MeSH] OR Maternal Health[MeSH] OR Pregnant[tiab] OR pregnancy[tiab] OR postpartum[tiab] OR antenatal[tiab] OR perinatal[tiab] OR postnatal[tiab] OR maternal[tiab] OR gestational[tiab] OR Pregnancy[MeSH] |
| 2 | Clinical protocols[mh] OR Consensus[mh] OR Consensus development conferences as topic[mh] OR Critical pathways[mh] OR Guidelines as topic [Mesh:NoExp] OR Practice guidelines as topic[mh] OR Health planning guidelines[mh] OR guideline[pt] OR practice guideline[pt] OR consensus development conference[pt] OR consensus development conference, NIH[pt] OR position statement*[tiab] OR policy statement*[tiab] OR practice parameter*[tiab] OR best practice*[tiab] OR standards[ti] OR guideline[ti] OR guidelines[ti] OR ((practice[tiab] OR treatment*[tiab]) AND guideline*[tiab]) OR CPG[tiab] OR CPGs[tiab] OR consensus*[tiab] OR ((critical[tiab] OR clinical[tiab] OR practice[tiab]) AND (path[tiab] OR paths[tiab] OR pathway[tiab] OR pathways[tiab] OR protocol*[tiab])) OR recommendat*[ti] OR (care[tiab] AND (standard[tiab] OR path[tiab] OR paths[tiab] OR pathway[tiab] OR pathways[tiab] OR map[tiab] OR maps[tiab] OR plan[tiab] OR plans[tiab])) OR (algorithm*[tiab] AND (screening[tiab] OR examination[tiab] OR test[tiab] OR tested[tiab] OR testing[tiab] OR assessment*[tiab] OR diagnosis[tiab] OR diagnoses[tiab] OR diagnosed[tiab] OR diagnosing[tiab])) OR (algorithm*[tiab] AND (pharmacotherap*[tiab] OR chemotherap*[tiab] OR chemotreatment*[tiab] OR therap*[tiab] OR treatment*[tiab] OR intervention*[tiab])) |
| 3 | Noncommunicable diseases[MeSH] OR chronic disease*[tiab] OR NCD[tiab] OR NCDs[tiab] Neoplasms[MeSH] OR cancer[tiab] OR Diabetes Mellitus[MeSH] OR diabetes[tiab] OR Hematologic Diseases[MeSH] OR thalassaemia[tiab] OR sickle cell[tiab] OR haemoglobinopath*[tiab] OR blood disorder*[tiab] OR immune disorder*[tiab] OR Mental disorders[MeSH] OR depressive[tiab] OR depression[tiab] OR bipolar[tiab] OR schizophrenia[tiab] OR suicid*[tiab] OR alcohol[tiab] OR substance use[tiab] OR substance abuse[tiab] OR drug use[tiab] OR anxiety[tiab] OR eating disorder*[tiab] OR autism[tiab] OR asperger[tiab] OR behavioural disorder[tiab] OR intellectual disability[tiab] OR dementia[MeSH] OR neurological[tiab] OR alzheimer[tiab] OR dementia[tiab] OR parkinson[tiab] OR epilepsy[tiab] OR multiple sclerosis[tiab] OR migraine[tiab] OR epilepsy[MeSH] OR Multiple Sclerosis[MeSH] OR Headache disorders[MeSH] OR Sensation Disorders[MeSH] OR Glaucoma[MeSH] OR glaucoma[tiab] OR cataract*[MeSH] OR refractive errors[MeSH] OR refractive errors[tiab] OR macular degeneration[MeSH] OR macular degeneration[tiab] OR vision loss[tiab] OR hearing loss[tiab] OR hearing loss[MeSH] OR cardiovascular diseases[MeSH] OR cardiovascular disease*[tiab] OR rheumatic heart disease[tiab] OR heart disease[tiab] OR stroke[tiab] OR cardiomyopathy[tiab] OR myocarditis[tiab] OR endocarditis[tiab] OR respiratory disease*[tiab] OR chronic obstructive pulmonary disease[tiab] OR COPD[tiab] OR asthma[tiab] OR sleep apnea[tiab] OR Respiratory Tract Diseases[MeSH] OR Digestive System Disease[MeSH] OR peptic ulcer disease[tiab] or cirrhosis[tiab] OR appendicitis[tiab] OR gastritis[tiab] OR duodenitis[tiab] OR ileus[tiab] OR intestinal obstruction[tiab] OR inflammatory bowel disease[tiab] OR gallbladder and biliary disease*[tiab] OR pancreatitis[tiab] OR Female Urogenital diseases[MeSH] OR Kidney diseases[MeSH] Or kidney disease*[tiab] OR urolithiasis[tiab] OR urolithiasis[MeSH] OR gynecological disease*[tiab] OR Skin diseases[MeSH] OR skin disease*[tiab] OR musculoskeletal diseases[MeSH] OR musculoskeletal disease*[tiab] OR rheumatoid arthritis[tiab] OR osteoarthritis[tiab] OR gout[tiab] OR back and neck pain[tiab] OR Congenital abnormalities[MeSH] OR congenital anomalities[tiab] OR congenital anomalies[tiab] OR neural tube defect*[tiab] OR cleft lip[tiab] OR cleft palate[tiab] OR down syndrome[tiab] OR chromosomal anomal*[tiab] OR congenital heart anomal*[tiab] OR Mouth Diseases[MeSH] OR dental caries[tiab] OR periodontal disease[tiab] OR edentulism[tiab] OR oral disorder*[tiab] OR oral condition*[tiab] |
| 4 | 1 AND 2 AND 3 from 2011/1/1 - 2021/5/31 |

**Table 2. Search strategy for Global Index Medicus^*^**

| **Search number** | **Query** |
| --- | --- |
| 1 | “PREGNANT” OR “POSTPARTUM” OR “PRENATAL” OR “PRENATAL CARE” OR “PERINATAL” OR “PERINATAL CARE” OR “MATERNAL” OR “PREGNANCY” OR “ANTENATAL” OR “ANTENATAL CARE” OR POSTNATAL” OR “POSTNATAL CARE” OR “GESTATIONAL” |
| 2 | (neoplasm) OR (cancer) OR (diabetes mellitus) OR (gestational diabetes) OR (diabetes) OR (hematologic disease) OR (hematologic disorder) OR (thalassaemia) OR (sickle cell) OR (haemoglobinopathy) OR (blood disorder) OR (immune disorder) OR (mental disorder) OR (depressive) OR (depression) OR (bipolar) OR (schizophrenia) OR (alcohol) OR (substance use) OR (drug use) OR (anxiety) OR (eating disorder) OR (autism) OR (asperger) OR (behavioural disorder) OR (intellectual disability) OR (dementia) OR (neurological) OR (alzheimer) OR (Parkinson) OR (epilepsy) OR (multiple sclerosis) OR (migraine) OR (headache) OR (sensation disorder) OR (glaucoma) OR (cataract) OR (refractive error) OR (macular degeneration) OR (vision loss) OR (hearing loss) OR (cardiovascular disease) OR (rheumatic heart disease) OR (heart disease) OR (stroke) OR (cardiomyopathy) OR (myocarditis) OR (endocarditis) OR (respiratory disease) OR (chronic obstructive pulmonary disease) OR (copd) OR (asthma) OR (respiratory tract disease) OR (digestive system disease) OR (peptic ulcer) OR (cirrhosis) OR (appendicitis) OR (gastritis) OR (duodenitis) OR (ileus) OR (intestinal obstruction) OR (inflammatory bowel disease) OR (gallbladder disease) OR (biliary disease) OR (pancreatitis) OR (urogenital disease) OR (urolithiasis) OR (gynecological disease) OR (skin disease) OR (musculoskeletal disease) OR (rheumatoid arthritis) OR (osteoarthritis) OR (gout) OR (back pain) OR (neck pain) OR (congenital abnormalities) OR (congenital anomalies) OR (neural tube defect) OR (cleft lip) OR (cleft palate) OR (down syndrome) OR (chromosomal anomalies) OR (congenital heart) OR (mouth disease) OR (dental caries) OR (periodontal disease) OR (edentulism) OR (oral disorder) OR (oral condition) |
| 3 | 1 AND 2 AND Filter for: Type of study (Practice guideline), Publication year (2011 to 2021) |

^*^ Including LILACS, WPRIM, IMSEAR, IMEMR, AIM databases

**Table 3. Search strategy for TRIP database**

| **Search number** | **Query** |
| --- | --- |
| 1 | (pregnant OR postpartum OR prenatal OR prenatal care OR perinatal OR perinatal care OR maternal OR pregnancy OR antenatal OR antenatal care OR postnatal OR postnatal care OR gestational) AND (title: noncommunicable OR non-communicable OR NCD OR NCDs OR preexisting OR pre-existing OR neoplasm OR cancer OR diabetes OR hematologic OR thalassaemia OR sickle cell OR haemoglobinopathy OR blood disorder OR immune disorder OR mental disorder OR depressive OR depression OR bipolar OR schizophrenia OR alcohol OR substance use OR drug use OR anxiety OR eating disorder OR autism OR asperger OR behavioural disorder OR intellectual disability OR dementia OR neurological OR alzheimer OR parkinson OR epilepsy OR multiple sclerosis OR migraine OR headache OR sensation disorder OR glaucoma OR cataract OR refractive error OR macular degeneration OR vision loss OR hearing loss OR cardiovascular disease OR rheumatic heart disease OR heart disease OR stroke OR cardiomyopathy OR myocarditis OR endocarditis OR respiratory disease OR sleep apnea OR chronic obstructive pulmonary disease OR COPD OR asthma OR respiratory tract disease) |
| 2 | 1 AND Filter for: Evidence Type (Guidelines), Publication year (2011 to 2021) |
