# Supplementary material for: Prevention and control of non-communicable diseases in antenatal, intrapartum, and postnatal care: a systematic scoping review of clinical practice guidelines since 2011
Source: BMC Med. 2022 Sep 20;20:305. doi: 10.1186/s12916-022-02508-9 (PMC9487084; doi:10.1186/s12916-022-02508-9)
Supplement: Supplementary file 4 — Additional file 4: Table 1. Websites of organizations, societies, associations, and colleges identified for searching of relevant guidelines. [file 12916_2022_2508_MOESM4_ESM.docx]

**Additional file 4. Prevention and control of non-communicable diseases in antenatal, intrapartum, and postpartum care: a systematic scoping review of clinical practice guidelines since 2011**

**Table 1. Websites of organizations, societies, associations, and colleges identified for searching of relevant guidelines**

| **Obstetrics and gynecology organizations and societies**  A total of 137 organizations and national societies were identified from the International Federation of Gynecology and Obstetrics list of member societies available here: https://www.figo.org/figos-member-societies. Websites, if available, were searched for relevant guidelines. | |
| --- | --- |
| **Medical colleges and associations related to non-communicable diseases**  The websites of the following medical colleges and associations relevant to non-communicable disease management were searched for relevant guidelines. | |
| **Country or region** | **Name of college or association** |
| United States of America | American College of Cardiology |
| United States of America | American College of Chest Physicians |
| Australia | Australasian College of Dermatologists |
| United States of America | American College of Emergency Physicians |
| United States of America | American College of Gastroenterology |
| United States of America | American College of Physicians |
| United States of America | American College of Preventive Medicine |
| United Kingdom | British Association of Dermatologists |
| Canada | Canadian Cardiovascular Society |
| Canada | Canadian Medical Association |
| Europe | European Association for Cardio-Thoracic |
| Europe | European Association of Neurology |
| Europe | European Hematology Association |
| Europe | European Society for Medical Oncology |
| Europe | European Society of Cardiology |
| Europe | European Society of Endocrinology |
| Europe | European Society of Gynaecological Oncology |
| Europe | European Society of Clinical Nutrition and Metabolism |
| Australia | Royal Australian College of General Practitioners |
| Australia/New Zealand | The Royal Australian & New Zealand College of Psychiatrists |
| United Kingdom | Royal College of General Practitioners |
| United Kingdom | Royal College of Physicians |
| United Kingdom | The Royal College of Paediatrics and Child Health |
| United States of America | U.S. Preventive Services Task Force |
| International | World Federation of Societies of Biological Psychiatry |
| Europe | European Respiratory Society |
| Europe | European Society for Vascular Surgery |
| Europe | European Society of Hypertension |
