# Supplementary material for: Prevention and control of non-communicable diseases in antenatal, intrapartum, and postnatal care: a systematic scoping review of clinical practice guidelines since 2011
Source: BMC Med. 2022 Sep 20;20:305. doi: 10.1186/s12916-022-02508-9 (PMC9487084; doi:10.1186/s12916-022-02508-9)
Supplement: Supplementary file 5 — Additional file 5: Table 1. Characteristics of identified guidelines for the management of non-communicable diseases during antenatal, intrapartum, and postnatal care by disease category. [file 12916_2022_2508_MOESM5_ESM.docx]

**Additional file 5. Prevention and control of non-communicable diseases in antenatal, intrapartum, and postpartum care: a systematic scoping review of clinical practice guidelines since 2011**

**Table 1. Characteristics of identified guidelines for the management of non-communicable diseases during antenatal, intrapartum, and postnatal care by disease category.**

| **Author(s)** | **Title** | **Year** | **Country/region** | **Pregnancy-specific** | **Condition(s) covered** |
| --- | --- | --- | --- | --- | --- |
| Cardiovascular disease | | | | | |
| European Society of Cardiology | 2011 European guidelines for the management of cardiovascular diseases during pregnancy. Part II: management of hypertension | 2011 | Europe | No | General cardiovascular diseases |
| European Society of Cardiology | ESC Guidelines on the management of cardiovascular diseases during pregnancy: the Task Force on the Management of Cardiovascular Diseases during Pregnancy of the European Society of Cardiology (ESC) | 2011 | Europe | Yes | Coronary heart disease and pulmonary hypertension, aortic disease; valvular heart disease, coronary artery disease; acute coronary syndrome; cardiomyopathies; heart failure; arrhythmias; hypertension |
| American Thoracic Society/ Society of Thoracic Radiology | An official American Thoracic Society/Society of Thoracic Radiology clinical practice guideline: evaluation of suspected pulmonary embolism in pregnancy | 2011 | United States of America | Yes | Pulmonary heart disease and diseases of pulmonary circulation |
| European Society of Cardiology | 2011 European Society of Cardiology guidelines for the management of cardiovascular diseases during pregnancy. Part 1 | 2011 | Europe | No | General cardiovascular diseases |
| Spanish Society of Neurosurgery | Aneurysmal subarachnoid hemorrhage: group of study of cerebrovascular pathology of the Spanish society of neurosurgery management guideline | 2011 | Spain | No | Stroke |
| American Heart Association | The American Heart Association 2010 guidelines for the management of cardiac arrest in pregnancy: consensus recommendations on implementation strategies | 2011 | United States of America | Yes | Other circulatory diseases |
| American Heart Association/ American Stroke Association | Guidelines for the prevention of stroke in patients with stroke or transient ischemic attack: a guideline for healthcare professionals from the American heart association/American stroke association | 2011 | United States of America | No | Stroke |
| American College of Cardiology Foundation / American Heart Association | 2011 ACCF/AHA Guideline for the diagnosis and treatment of hypertrophic cardiomyopathy | 2011 | United States of America | No | Cardiomyopathy/myocarditis/endocarditis |
| European Society of Cardiology | Cardiovascular diseases during pregnancy | 2011 | Europe | Yes | Cardiomyopathy/myocarditis/endocarditis; Ischemic heart disease; Rheumatic heart disease; Other circulatory diseases |
| Polish Society of Arterial Hypertension | Treatment of arterial hypertension in pregnancy in relation to current guidelines of the Polish Society of Arterial Hypertension from 2011 | 2012 | Poland | Yes | Hypertensive heart disease |
| American College of Chest Physicians | Diagnosis of DVT: Antithrombotic Therapy and Prevention of Thrombosis, 9th ed: American College of Chest Physicians Evidence-Based Clinical Practice Guidelines | 2012 | United States of America | No | Other circulatory diseases |
| Spanish Society of Pneumology and Thoracic Surgery | National Consensus on the Diagnosis, Risk Stratification and Treatment of Patients with Pulmonary Embolism. Spanish Society of Pneumology and Thoracic Surgery (SEPAR). Society Española Internal Medicine (SEMI). Spanish Society of Thrombosis and Haemostasis (SETH). Spanish Society of Cardiology (ESC). Spanish Society of Medicine Accident and Emergency (SEMES). Spanish Society of Angiology and Surgery Vascular (SEACV) | 2013 | Spain | No | Other circulatory diseases |
| European Society of Hypertension / European Society of Cardiology | 2013 ESH/ESC Guidelines for the management of arterial hypertension | 2013 | Europe | No | Hypertensive heart disease |
| European Society of Cardiology / European Heart Rhythm Association | 2013 ESC Guidelines on cardiac pacing and cardiac resynchronization therapy: the Task Force on cardiac pacing and resynchronization therapy of the European Society of Cardiology (ESC). Developed in collaboration with the European Heart Rhythm Association (EHRA) | 2013 | Europe | No | Other circulatory diseases |
| Society of Obstetric Medicine Australian and NZ | The SOMANZ Guidelines for the Management of Hypertensive Disorders of Pregnancy 2014 | 2014 | Australia, New Zealand | Yes | Hypertensive disorders of pregnancy |
| Society for Obstetric Anesthesia and Perinatology | The Society for Obstetric Anesthesia and Perinatology consensus statement on the management of cardiac arrest in pregnancy | 2014 | United States of America | Yes | Other circulatory diseases |
| European Society of Cardiology | 2014 ESC guidelines on diagnosis and management of pulmonary embolism | 2014 | United States of America | No | Other circulatory diseases |
| European Society of Cardiology | 2014 ESC guidelines on diagnosis and management of hypertrophic cardiomyopathy: The Task Force for the diagnosis and management of hypertrophic cardiomyopathy of the European Society of Cardiology (ESC) | 2014 | United States of America | No | Cardiomyopathy/myocarditis/endocarditis |
| European Society of Cardiology | Hypertrophic Cardiomyopathy | 2014 | Europe | No | Cardiomyopathy/myocarditis/endocarditis |
| American Heart Association / American College of Cardiology | 2014 AHA/ACC Guideline for the management of patients with non-ST elevation acute coronary syndromes | 2014 | United States of America | No | Ischemic heart disease |
| American Heart Association / American College of Cardiology | 2014 AHA/ACC Guideline for the management of patients with valvular heart disease | 2014 | United States of America | No | Rheumatic heart disease |
| American Heart Association / American Stroke Association | Guidelines for the Prevention of Stroke in Women A Statement for Healthcare Professionals from the American Heart Association/American Stroke Association | 2014 | United States of America | No | Stroke |
| Ministry of Labour, Health and Social Affairs of Georgia | Cardiovascular disease management during pregnancy | 2014 | Georgia | Yes | General cardiovascular diseases |
| American College of Cardiology / American Heart Association /Heart Rhythm Society | 2015 ACC/AHA/HRS Guideline for the management of adult patients with supraventricular tachycardia | 2015 | United States of America | No | Other circulatory diseases |
| European Society of Cardiology | 2015 ESC Guidelines for the management of patients with ventricular arrhythmias and the prevention of sudden cardiac death | 2015 | Europe | No | Other circulatory diseases |
| American Heart Association | Cardiac Arrest in Pregnancy: A Scientific Statement from the American Heart Association | 2015 | United States of America | Yes | Other circulatory diseases |
| French Society of Hypertension | Hypertension and pregnancy. Expert consensus statement from the French Society of Hypertension, an affiliate of the French Society of Cardiology | 2016 | France | Yes | Hypertensive disorders of pregnancy |
| Chinese Medical Association | Expert consensus document of the diagnosis and treatment of pregnancy with heart disease | 2016 | China | Yes | General cardiovascular diseases |
| Government of South Australia | Cardiac disease in pregnancy | 2016 | Australia | Yes | Cardiomyopathy, myocarditis, endocarditis; Ischemic heart disease; Rheumatic heart disease; Other circulatory diseases |
| Institute for Clinical Systems Improvement | Diagnosis and Initial Treatment of Ischemic Stroke | 2016 | United States of America | No | Stroke |
| Ministry of Health /AMM/ National Heart Association Malaysia | Heart Disease in Pregnancy 2nd Edition | 2016 | Malaysia | Yes | General cardiovascular diseases |
| European Society of Cardiology / The European Association for Cardio-Thoracic Surgery | 2016 ESC Guidelines for the management of atrial fibrillation developed in collaboration with EACTS | 2016 | Europe | No | Other circulatory diseases |
| Canadian Cardiovascular Society | 2017 Comprehensive Update of the Canadian Cardiovascular Society Guidelines for the Management of Heart Failure | 2017 | Canada | No | Other circulatory diseases |
| American Heart Association | Pregnancy, Hormonal Treatments for Infertility, Contraception, and Menopause in Women After Ischemic Stroke A Consensus Document | 2017 | United States of America | No | Stroke |
| European Society of Cardiology | 2017 Antithrombotic Therapy in Atrial Fibrillation Associated with Valvular Heart Disease: A Joint Consensus Document from EHRA and ESC Working Group on Thrombosis | 2017 | Europe | No | Other circulatory diseases |
| European Society of Cardiology / The European Association for Cardio-Thoracic Surgery | 2017 ESC/EACTS Guidelines for the management of valvular heart disease | 2017 | Europe | No | Rheumatic heart disease |
| Society of Cardiovascular Computed Tomography | Coronary computed tomographic imaging in women: An expert consensus statement from the Society of Cardiovascular Computed Tomography | 2018 | International | No | Ischemic heart disease |
| European Society of Cardiology | 2018 ESC Guidelines for the management of cardiovascular diseases during pregnancy | 2018 | Europe | Yes | Cardiomyopathy, myocarditis, endocarditis; Ischemic heart disease; Other circulatory diseases |
| Expert consensus | Canadian Stroke Best Practice Consensus Statement: Acute Stroke Management during pregnancy | 2018 | Canada | Yes | Stroke |
| American Heart Association / American College of Cardiology | 2017 AHA/ACC/HRS Guideline for Management of Patients With Ventricular Arrhythmias and the Prevention of Sudden Cardiac Death: A Report of the American College of Cardiology/American Heart Association Task Force on Clinical Practice Guidelines and the Heart Rhythm Society | 2018 | United States of America | No | Other circulatory diseases |
| Hypertension Canada | Hypertension Canada's 2018 Guidelines for the Management of Hypertension in Pregnancy | 2018 | Canada | Yes | Hypertensive disorders of pregnancy |
| International Society for Study of Hypertension in Pregnancy | Hypertensive Disorders of Pregnancy: ISSHP Classification, Diagnosis, and Management Recommendations for International Practice | 2018 | International | Yes | Hypertensive disorders of pregnancy |
| Ministry of Health (Kenya) | Kenya national guidelines for cardiovascular diseases management | 2018 | Kenya | No | Hypertensive disorders of pregnancy;  Other circulatory diseases |
| European Society of Cardiology / European Society of Hypertension | 2018 ESC/ESH Guidelines for the management of arterial hypertension | 2018 | Europe | No | Hypertensive heart disease |
| Ministerial National Committee on Confidential Enquiries into Maternal Deaths in South Africa | Hypertensive disorders in pregnancy: 2019 National guidelines | 2019 | South Africa | Yes | Hypertensive heart disease |
| Polish Society of Hypertension / Polish Cardiac Society / Polish Society of Gynecologists and Obstetricians | Management of hypertension in pregnancy: prevention, diagnosis, treatment and long-term prognosis | 2019 | Poland | Yes | Hypertensive heart disease |
| American College of Obstetricians and Gynecologists | ACOG Practice Bulletin No. 203: Chronic Hypertension in Pregnancy | 2019 | United States of America | Yes | Hypertensive heart disease |
| American College of Obstetricians and Gynecologists | ACOG Practice Bulletin No. 212: Pregnancy and Heart Disease | 2019 | United States of America | Yes | General cardiovascular diseases |
| American College of Obstetricians and Gynecologists | Practice Bulletin No. 212: Pregnancy and Heart Disease | 2019 | United States of America | Yes | General cardiovascular diseases |
| Royal College of Physicians of Ireland | The management of hypertension in pregnancy | 2019 | Ireland | Yes | Hypertensive heart disease |
| National Institute for Health and Care Excellence | Hypertension in pregnancy: diagnosis and management | 2019 | United Kingdom | Yes | Hypertensive heart disease |
| American Heart Association / American College of Cardiology | 2020 AHA/ACC Guideline for the Diagnosis and Treatment of Patients With Hypertrophic Cardiomyopathy: A Report of the American College of Cardiology/American Heart Association Joint Committee on Clinical Practice Guidelines | 2020 | United States of America | No | Cardiomyopathy/myocarditis/endocarditis |
| RHDAustralia | The 2020 Australian guideline for prevention, diagnosis and management of acute rheumatic fever and rheumatic heart disease | 2020 | Australia | No | Rheumatic heart disease |
| Hypertension Canada | Hypertension Canada's 2020 Comprehensive Guidelines for the Prevention, Diagnosis, Risk Assessment, and Treatment of Hypertension in Adults and Children | 2020 | Canada | No | Hypertensive heart disease |
| European Society of Cardiology | 2019 ESC Guidelines for the diagnosis and management of acute pulmonary embolism developed in collaboration with the European Respiratory Society (ERS) | 2020 | Europe | No | Other circulatory diseases |
| Guidelines Working Group | Second International Guidelines for the Diagnosis and Management of Hereditary Hemorrhagic Telangiectasia | 2020 | International | No | Other circulatory diseases |
| American College of Cardiology Foundation | Summary of Updated Recommendations for Primary Prevention of Cardiovascular Disease in Women: JACC State-of-the-Art Review | 2020 | United States of America | No | General cardiovascular diseases |
| Chinese Medical Association | Diagnosis and treatment of hypertension and pre-eclampsia in pregnancy: a clinical practice guideline in China（2020) | 2020 | China | Yes | Hypertensive disorders of pregnancy |
| Brazilian Cardiology Society | Brazilian Cardiology Society Statement for Management of Pregnancy and Family Planning in Women with Heart Disease - 2020 | 2020 | Brazil | No | General cardiovascular diseases |
| Cardiac Society of Australia and New Zealand | Rheumatic fever and rheumatic heart disease in Australia (3rd Edition) | 2020 | Australia/  New Zealand | No | Rheumatic heart disease |
| The Royal Thai College of Obstetricians and Gynaecologists | Management of Hypertensive Disorders in Pregnancy | 2020 | Thailand | Yes | Hypertensive heart disease |
| European Society of Cardiology | 2019 ESC Guidelines for the management of patients with supraventricular tachycardia | 2020 | Europe | No | Other circulatory diseases |
| Canadian Cardiovascular Society | 2021 CCS Guidelines for the Management of Dyslipidaemia for the Prevention of Cardiovascular Disease in the Adult | 2021 | Canada | No | Other circulatory diseases |
| American College of Cardiology / American Heart Association | 2020 ACC/AHA Guidelines for the Management of Patients With Valvular Heart Disease | 2021 | United States of America | No | Rheumatic heart disease |
| The Swedish Society Obstetrics Gynecology | Riktlinjer för hypertonisjukdomar under graviditet | 2021 | Sweden | Yes | Hypertensive heart disease |
| **Malignant neoplasms** | | | | | |
| Japan Society of Gynecologic Oncology | Japan Society of Gynecologic Oncology guidelines 2011 for the treatment of uterine cervical cancer | 2011 | Japan | No | Cancer-Cervical |
| Expert consensus | Manejo del cáncer de recto durante el embarazo | 2011 | Chile | Yes | Cancer-Colon and rectum |
| Royal College of Obstetricians and Gynecologists | Pregnancy and Breast Cancer | 2011 | United Kingdom | Yes | Cancer-Breast |
| European Society of Breast Cancer Specialists | The European Society of Breast Cancer Specialists recommendations for the management of young women with breast cancer | 2012 | Europe | No | Cancer-Breast |
| American Society for Colposcopy and Cervical Pathology | 2012 Updated consensus guidelines for the management of abnormal cervical cancer screening tests and cancer precursors | 2012 | United States of America | No | Cancer-Cervical |
| Society of Obstetricians and Gynaecologists of Canada | Cancer chemotherapy and pregnancy | 2013 | Canada | Yes | General cancer |
| Korean Society of Gynecologic Oncology / Korean Society for Cytopathology | Practice guidelines for the early detection of cervical cancer in Korea: Korean Society of Gynecologic Oncology and the Korean Society for Cytopathology 2012 edition | 2013 | South Korea | No | Cancer-Cervical |
| European Association of Nuclear Medicine / The Society of Nuclear Medicine and Molecular Imaging | The EANM and SNMMI practice guideline for lymphoscintigraphy and sentinel node localization in breast cancer | 2013 | Europe | No | Cancer-Breast |
| European Society for Medical Oncology | Cancer, pregnancy and fertility: ESMO Clinical Practice Guidelines for diagnosis, treatment and follow-up | 2013 | Europe | Yes | General cancer |
| British Committee for Standards in Haematology | Guidelines for the first line management of classical Hodgkin lymphoma | 2014 | United Kingdom | No | Cancer-Lymphoma/multiple myeloma |
| French Society of Haematology | Recommendations of the SFH (French Society of Haematology) for the diagnosis, treatment and follow-up of hairy cell leukaemia | 2014 | France | No | Cancer-Leukemia |
| International Gynecologic Cancer Society/ European Society of Gynaecological Oncology | Gynecologic cancers in pregnancy: guidelines of a second international consensus meeting | 2014 | International | Yes | Gynecological cancer |
| College National des Gynecologues Obstetriciens Francais | Management of presumed benign ovarian tumors: updated French guidelines | 2014 | France | No | Cancer-Ovary |
| British Thyroid Association | Management of thyroid cancer | 2014 | United Kingdom | No | Cancer-Thyroid |
| European Association of Nuclear Medicine | The EANM clinical and technical guidelines for lymphoscintigraphy and sentinel node localization in gynaecological cancers | 2014 | Europe | No | Gynecological cancer; Cancer-Other |
| German Cancer Society / Union Internationale Contre le Cancer | Breast Cancer Diagnosed During Pregnancy: Adapting Recent Advances in Breast Cancer Care for Pregnant Patients | 2015 | Germany | Yes | Cancer-Breast |
| European Association of Nuclear Medicine | EANM practice guidelines for lymphoscintigraphy and sentinel lymph node biopsy in melanoma | 2015 | Europe | No | Cancer-Breast |
| World Allergy Organization | Comprehensive cervical cancer control | 2015 | International | No | Cancer-Cervical |
| American College of Radiology | Conservative surgery and radiation - Stage I and II Breast cancer | 2015 | United States of America | No | Cancer-Breast |
| Ministry of Health | Management of Cervical Cancer | 2015 | Malaysia | No | Cancer-Cervical |
| American Thyroid Association | 2015 American Thyroid Association Management Guidelines for Adult Patients with Thyroid Nodules and Differentiated Thyroid Cancer | 2015 | United States of America | No | Cancer-Thyroid |
| European Society for Medical Oncology | Philadelphia chromosome-negative chronic myeloproliferative neoplasms: ESMO Clinical Practice Guidelines for diagnosis, treatment and follow-up | 2015 | Europe | No | Other neoplasms |
| Danish Society of Obstetrics and Gynaecology | Cancer in pregnancy | 2015 | Denmark | Yes | General cancer |
| National Comprehensive Cancer Network | NCCN Guidelines Insights: Chronic Myeloid Leukemia, Version 1.2017 | 2016 | United States of America | No | Cancer-Leukemia |
| Ministry of Health | Cervical Cancer Screening: Recommendations for Muslim Societies | 2016 | Iran | No | Cancer-Cervical |
| German Diabetes Association | Surgical treatment of melanoma in pregnancy: a practical guideline | 2016 | Germany | Yes | Cancer-Melanoma |
| Ginekologia i Perinatologia Praktyczna | The conventional treatment of pregnant women with cancer | 2016 | Poland | Yes | General cancer |
| American Society of Clinical Oncology | ASCCP Colposcopy Standards: How Do We Perform Colposcopy? Implications for Establishing Standards | 2017 | United States of America | No | Cancer-Cervical |
| American College of Radiology | ACR Appropriateness Criteria Palpable Breast Masses | 2017 | United States of America | No | Cancer-Breast; Gynecological diseases |
| British Society for Haematology | A guideline for the management of specific situations in polycythaemia vera and secondary erythrocytosis: A British Society for Haematology Guideline | 2018 | United Kingdom | No | Other malignant neoplasms |
| Society of Obstetricians and Gynaecologists of Canada | No. 366-Gynaecologic Management of Hereditary Breast and Ovarian Cancer | 2018 | Canada | No | Cancer-Breast; Ovarian cancer |
| National Comprehensive Cancer Network | Adolescent and Young Adult Oncology, Version 2.2018, NCCN Clinical Practice Guidelines in Oncology | 2018 | United States of America | No | Other malignant neoplasms; Cancer-Other |
| ESCO/ European Society for Radiotherapy and Oncology/ European Society of Pathology | The European Society of Gynaecological Oncology/European Society for Radiotherapy and Oncology/European Society of Pathology Guidelines for the Management of Patients With Cervical Cancer | 2018 | Europe | No | Cancer-Cervical |
| American College of Radiology | ACR Appropriateness Criteria Breast Imaging of Pregnant and Lactating Women | 2018 | United States of America | Yes | Cancer-Breast |
| European Society of Endocrinology/ European Network for the Study of Adrenal Tumors | European Society of Endocrinology Clinical Practice Guidelines on the management of adrenocortical carcinoma in adults, in collaboration with the European Network for the Study of Adrenal Tumors | 2018 | Europe | No | Other malignant neoplasms; Cancer-Other |
| American Academy of Dermatology | Guidelines of care for the management of primary cutaneous melanoma | 2019 | United States of America | No | Cancer-Melanoma |
| European LeukemiaNet | Management of acute promyelocytic leukemia: updated recommendations from an expert panel of the European LeukemiaNet | 2019 | Multinational | No | Cancer-Leukemia |
| International Network on Cancer, Infertility and Pregnancy | Gynecologic cancers in pregnancy: guidelines based on a third international consensus meeting | 2019 | International | Yes | Gynecological cancer; Cancer-Other |
| Chinese Medical Association | Chinese consensus guidelines for breast cancer in young women: clinical practice and fertility preservation | 2019 | China | No | Cancer-Breast |
| American Society of Breast Surgeons | Consensus Guideline on the Management of the Axilla in Patients With Invasive/In-Situ Breast Cancer | 2019 | United States of America | No | Cancer-Breast |
| Cancer Council Australia | Cervical cancer Screening | 2019 | Australia | No | Cancer-Cervical |
| The Swedish Society Obstetrics Gynecology | SFOG-riktlinje av NVP för cervixcancerprevention | 2019 | Sweden | No | Cancer-Cervical |
| Societatea de Obstetrică şi Ginecologie din România şi Colegiul Medicilor din România | Cancerul de col uterin | 2019 | Romania | No | Cancer-Cervical |
| Cancer Council Australia | Melanoma | 2019 | Australia | No | Cancer-Melanoma |
| Society of Gynecologic Oncology | Diagnosis and Management of Adenocarcinoma in Situ: A Society of Gynecologic Oncology Evidence-Based Review and Recommendations | 2020 | United States of America | No | Other neoplasms |
| European Society for Medical Oncology | Fertility preservation and post-treatment pregnancies in post-pubertal cancer patients: ESMO Clinical Practice Guidelines | 2020 | Europe | Yes | Other neoplasms |
| LeukemiaNet | European LeukemiaNet 2020 recommendations for treating chronic myeloid leukemia | 2020 | Europe | No | Cancer-Leukemia |
| Cancer Australia | Guidance for the management of early breast cancer: Recommendations and practice points | 2020 | Australia | No | Cancer-Breast |
| American Society for Clinical Pathology | 2019 ASCCP Risk-Based Management Consensus Guidelines for Abnormal Cervical Cancer Screening Tests and Cancer Precursors | 2020 | United States of America | No | Cancer-Cervical |
| British Gynaecological Cancer Society | British Gynaecological Cancer Society (BGCS) cervical cancer guidelines: Recommendations for practice | 2021 | United Kingdom | No | Cancer-Cervical |
| European Federation for Colposcopy and Pathology of the Lower Genital Tract / European Society of Gynaecological Oncology | European consensus statement on essential colposcopy | 2021 | Europe | No | Cancer-Cervical |
| College National des Gynecologues Obstetriciens Francais | Borderline ovarian tumors: Guidelines from the French national college of obstetricians and gynecologists (CNGOF) | 2021 | France | No | Cancer-Ovary |
| **Congenital anomalies** | | | | | |
| European Society of Cardiology | Cardiovascular diseases during pregnancy | 2011 | Europe | Yes | Congenital heart anomalies |
| American Urological Association Working Group | The Recommendations of the 2015 American Urological Association Working Group on Genitourinary Congenitalism | 2016 | United States of America | No | Other congenital anomalies |
| British Thoracic Society | British Thoracic Society Clinical Statement on Pulmonary Arteriovenous Malformations | 2017 | United Kingdom | No | Congenital heart anomalies |
| Italian Association of Hospital Cardiologists/ Italian Society of Pediatric Cardiology / Italian Society of Gynaecologists and Obstetrics | ANMCO/SICP/SIGO Consensus document: Pregnancy and congenital heart disease | 2017 | Italy | Yes | Congenital heart anomalies |
| International Turner Syndrome Consensus Group | Clinical practice guidelines for the care of girls and women with Turner syndrome: proceedings from the | 2017 | International | No | Other chromosomal anomalies |
| World Allergy Organization / European Academy of Allergy and Clinical Immunology | The international WAO/EAACI guideline for the management of hereditary angioedema-The 2017 revision and update | 2017 | International | No | Other congenital anomalies |
| American Heart Association | Cardiovascular Health in Turner Syndrome: A Scientific Statement From the American Heart Association | 2018 | United States of America | No | Other chromosomal anomalies |
| Skeletal Dysplasia Management Consortium | Best practice guidelines regarding prenatal evaluation and delivery of patients with skeletal dysplasia | 2018 | United States of America | Yes | Other congenital anomalies |
| American College of Cardiology/ American Heart Association | Adults With Congenital Heart Disease | 2018 | United States of America | No | Congenital heart anomalies |
| French Society of Otorhinolaryngology | Guidelines (short version) of the French Society of Otorhinolaryngology (SFORL) on cervical lymphatic malformation in adults and children: Diagnosis | 2019 | France | No | Other congenital anomalies |
| **Diabetes mellitus** | | | | | |
| Expert committee | Guidelines and recommendations for laboratory analysis in the diagnosis and management of diabetes mellitus | 2011 | United States of America | No | Diabetes-Gestational |
| The Royal College of Ophthalmologists | Diabetic Retinopathy Guidelines | 2012 | United Kingdom | No | Diabetes-Type1/2 |
| Polish Society of Endocrinology /Polish Diabetes Association | Recommendations of the Polish Society of Endocrinology and Polish Diabetes Association for the management of thyroid dysfunction in type 1 and type 2 diabetes | 2013 | Poland | No | Diabetes-Type1/2 |
| Endocrine Society | Diabetes and pregnancy: an endocrine society clinical practice guideline | 2013 | International | Yes | Diabetes-Gestational |
| Global Partnership for Effective Diabetes Management | Individualized glycemic targets and pharmacotherapy in type 2 diabetes | 2013 | International | No | Diabetes-Type1/2 |
| Canadian Diabetes Association | Canadian Diabetes Association 2013 clinical practice guidelines for the prevention and management of diabetes in Canada | 2013 | Canada | No | Diabetes-Type1/2/Gestational |
| Expert consensus | Consensus evidence-based guidelines for management of gestational diabetes mellitus in India | 2014 | India | Yes | Diabetes-Gestational |
| U.S. Preventive Services Task Force | Screening for gestational diabetes mellitus: U.S. Preventive Services Task Force recommendation statement | 2014 | United States of America | Yes | Diabetes-Gestational |
| German Diabetes Association/ German Association for Gynaecology and Obstetrics | Gestational diabetes mellitus (GDM) diagnosis, therapy and follow-up care: Practice Guideline of the German Diabetes Association(DDG) and the German Association for Gynaecology and Obstetrics (G) | 2014 | Germany | Yes | Diabetes-Gestational |
| World Health Organization | Diagnostic criteria and classification of hyperglycaemia first detected in pregnancy: a World Health Organization Guideline | 2014 | International | Yes | Diabetes-Gestational |
| Chinese Medical Association | Diagnosis and therapy guideline of pregnancy with diabetes mellitus | 2014 | China | Yes | Diabetes-Gestational |
| Polish Gynecologically Society | Actualisation of Polish Gynecological Society standards of medical care in management of women with diabetes | 2014 | Poland | No | Diabetes-Gestational |
| Ministry of Health | Screening, diagnosis and management of gestational diabetes in New Zealand: a clinical practice guideline | 2014 | New Zealand | Yes | Diabetes-Gestational |
| Chilena de Endocrinología y Diabetes | Second Consensus of the Chilean Society of Endocrinology and Diabetes about insulin resistance | 2015 | Chile | No | Diabetes-Type1/2 |
| The International Federation of Gynecology and Obstetrics | The International Federation of Gynecology and Obstetrics (FIGO) Initiative on gestational diabetes mellitus: A pragmatic guide for diagnosis, management, and care | 2015 | International | Yes | Diabetes-Gestational |
| Hospital Clínico Universidad de Chile | Serie guías clínicas: diabetes y embarazo (1ª parte): cuidado preconcepcional, diagnóstico y seguimiento | 2015 | Chile | Yes | Diabetes-Gestational |
| American Association of Clinical Endocrinologists/ American College of Endocrinology | AACE/ACE Clinical Practice Guidelines for Developing a Diabetes Mellitus Comprehensive Care Plan | 2015 | United States of America | No | Diabetes-Type1/2 |
| National Institute for Health and Care Excellence | Diabetes in pregnancy: management from preconception to the postnatal period | 2015 | United Kingdom | Yes | Diabetes-Type1/2/Gestational |
| Ministry of Labour, Health and Social Affairs of Georgia | Management of gestational diabetes (გესტაციური დიაბეტის მართვა) | 2015 | Georgia | Yes | Diabetes-Gestational |
| Asociación Latinoamericana de Diabetes | Guías de diagnóstico y tratamiento de la diabetes gestacional. ALAD, 2016 | 2016 | Latin America | Yes | Diabetes-Gestational |
| Royal Australian College of General Practitioners | General practice management of type 2 diabetes 2016-2018 | 2016 | Australia | No | Diabetes-Type1/2/Gestational |
| Expert consensus | Consensus on "Basal insulin in the management of Type 2 Diabetes: Which, When and How?" | 2017 | India | No | Diabetes-Type1/2 |
| Association of British Clinical Diabetologists | Management of glycaemic control in pregnant women with diabetes on obstetric wards and delivery units | 2017 | United Kingdom | Yes | Diabetes-Type1/2/Gestational |
| The Royal Thai College of Obstetricians and Gynaecologists | Diabetes Mellitus Screening in Pregnancy | 2017 | Thailand | Yes | Diabetes-Gestational |
| Ministry of Health (Malaysia)/ Malaysian Endocrine and Metabolic Society / Perinatal Society of Malaysia / Family Medicine Specialists Association of Malaysia / Academy of Medicine Malaysia | Management of Diabetes in Pregnancy | 2017 | Malaysia | Yes | Diabetes-Gestational |
| International Council of Ophthalmology | Guidelines on Diabetic Eye Care: The International Council of Ophthalmology Recommendations for Screening, Follow-up, Referral, and Treatment Based on Resource Settings | 2018 | International | No | Diabetes-Type1/2 |
| Polish Gynecological Society | Polish Gynecological Society standards of medical care in management of women with diabetes | 2018 | Poland | Yes | Diabetes-Type1/2/Gestational |
| Diabetes UK | Academy of Nutrition and Dietetics Gestational Diabetes Evidence-Based Nutrition Practice Guideline | 2018 | United Kingdom | No | Diabetes-Gestational |
| Academy of Nutrition and Dietetics | Academy of Nutrition and Dietetics Gestational Diabetes Evidence-Based Nutrition Practice Guideline | 2018 | United States of America | Yes | Diabetes-Gestational |
| American College of Obstetricians and Gynecologists | ACOG Practice Bulletin No. 190: Gestational Diabetes Mellitus | 2018 | United States of America | Yes | Diabetes-Gestational |
| American College of Obstetricians and Gynecologists | ACOG Practice Bulletin No. 201: Pregestational Diabetes Mellitus | 2018 | United States of America | Yes | Diabetes-Gestational |
| Diabetes Canada | Clinical Practice Guidelines for the Prevention and Management of Diabetes in Canada | 2018 | Canada | No | Diabetes-Type1/2/Gestational |
| Society of Obstetricians and Gynaecologists of Canada | Guideline No. 393-Diabetes in Pregnancy | 2019 | Canada | Yes | Diabetes-Gestational |
| Universidade Federal Do Rio Grande Do Sul | TeleCondutas: diabetes e gestação | 2019 | Brazil | No | Diabetes-Gestational |
| Government of South Australia | Gestational diabetes and diabetes mellitus | 2019 | Australia | Yes | Diabetes-Type1/2/Gestational |
| German Diabetes Association | Diabetes und Schwangerschaft | 2019 | Germany | No | Diabetes-Type1/2 |
| American Diabetes Association | Standards of Medical Care in Diabetes | 2021 | United States of America | No | Diabetes-Type1/2/Gestational |
| **Digestive diseases** | | | | | |
| Italian Society of Gastroenterology/ Italian Group for the study of Inflammatory Bowel Disease | The Italian Society of Gastroenterology (SIGE) and the Italian Group for the study of Inflammatory Bowel Disease (IG-IBD) Clinical Practice Guidelines: The use of tumor necrosis factor-alpha antagonist therapy in inflammatory bowel disease | 2011 | Italy | No | Inflammatory bowel disease |
| Society for Maternal-Fetal Medicine / American College of Obstetricians and Gynecologists | Society for Maternal-Fetal Medicine Consult Series #53: Intrahepatic cholestasis of pregnancy: Replaces Consult #13, April 2011 | 2011 | United States of America | Yes | Gallbladder and biliary diseases |
| Canadian Association of Gastroenterology | Consensus statements on the risk, prevention, and treatment of venous thromboembolism in inflammatory bowel disease: Canadian Association of Gastroenterology | 2014 | Canada | No | Inflammatory bowel disease |
| European Crohn's and Colitis Organization | The second European evidenced-based consensus on reproduction and pregnancy in inflammatory bowel disease | 2015 | Europe | Yes | Inflammatory bowel disease |
| Italian Society of Colorectal Surgery | Evaluation and management of hemorrhoids: Italian society of colorectal surgery (SICCR) consensus statement | 2015 | Italy | No | Other digestive diseases |
| Kidney Health Australia | KHA-CARI guideline: Early chronic kidney disease: detection, prevention and management | 2015 | Australia | No | Kidney diseases |
| The Italian Surgical Societies Working Group | Laparoscopic cholecystectomy: consensus conference-based guidelines | 2015 | Italy | No | Gallbladder and biliary diseases |
| Korean Association for the Study of Intestinal Diseases | Use of Thiopurines in Inflammatory Bowel Disease: A Consensus Statement by the Korean Association for the Study of Intestinal Diseases (KASID) | 2015 | South Korea | No | Inflammatory bowel disease |
| Pan American Crohn's and Colitis Organization | Special situations in inflammatory bowel disease: First Latin American consensus of the Pan American Crohn's and Colitis Organisation (PANCCO) (Second part) | 2016 | Latin America | No | Inflammatory bowel disease |
| The American College of Gastroenterology | ACG Clinical Guideline: Liver Disease and Pregnancy | 2016 | United States of America | Yes | Other digestive diseases |
| Crohn's & Colitis Foundation of America | Experts Opinion on the Practical Use of Azathioprine and 6-Mercaptopurine in Inflammatory Bowel Disease | 2016 | United States of America | No | Inflammatory bowel disease |
| IBD in Pregnancy Consensus Group | The Toronto Consensus Statements for the Management of Inflammatory Bowel Disease in Pregnancy | 2016 | Canada | Pregnancy-specific | Inflammatory bowel disease |
| Mexicana de Gastroenterologia | Diagnosis and treatment of gastroesophageal reflux disease: recommendations of the Asociación Mexicana de Gastroenterología | 2016 | Mexico | No | Other digestive diseases |
| The Italian Association for the Study of the Liver | ASIF position paper on liver disease and pregnancy | 2016 | Italy | Pregnancy-specific | Other digestive diseases |
| Chinese Medical Association | Consensus on the diagnosis and treatment of cholestatic liver diseases (2015, China) | 2016 | China | No | Gallbladder and biliary diseases |
| European Crohn's and Colitis Organization | Third European Evidence-based Consensus on Diagnosis and Management of Ulcerative Colitis. Part 1: Definitions, Diagnosis, Extra-intestinal Manifestations, Pregnancy, Cancer Surveillance, Surgery, and Ileo-anal Pouch Disorders | 2017 | Europe | No | Inflammatory bowel disease |
| Italian Group for the study of Inflammatory Bowel Disease | Safety of treatments for inflammatory bowel disease: Clinical practice guidelines of the Italian Group for the Study of Inflammatory Bowel Disease (IG-IBD) | 2017 | Italy | No | Inflammatory bowel disease |
| European Society for Clinical Nutrition and Metabolism | ESPEN guideline: Clinical nutrition in inflammatory bowel disease | 2017 | Europe | No | Inflammatory bowel disease |
| Spanish Working Group on Crohn's Disease and Ulcerative Colitis | Recommendations of the Spanish Working Group on Crohn's Disease and Ulcerative Colitis (GETECCU) on the use of thiopurines in inflammatory bowel disease | 2018 | Spain | No | Inflammatory bowel disease |
| The Asian Working Group | Diet and inflammatory bowel disease: The Asian Working Group guidelines | 2019 | Multinational | No | Inflammatory bowel disease |
| American Gastroenterological Association | Inflammatory Bowel Disease in Pregnancy Clinical Care Pathway: A Report From the American Gastroenterological Association IBD Parenthood Project Working Group | 2019 | United States of America | Pregnancy-specific | Inflammatory bowel disease |
| British Society of Gastroenterology | British Society of Gastroenterology consensus guidelines on the management of inflammatory bowel disease in adults | 2019 | United Kingdom | No | Inflammatory bowel disease |
| Chinese Medical Association | Expert recommendations on standardized diagnosis and treatment for fatty liver disease in China (2019 revised edition) | 2019 | China | No | Other digestive diseases |
| Italian Group for the study of Inflammatory Bowel Disease | Are we choosing wisely for inflammatory bowel disease care? The IG-IBD choosing wisely campaign | 2020 | Italy | No | Inflammatory bowel disease |
| European Hernia Society /Americas Hernia Society | EHS and AHS guidelines for treatment of primary ventral hernias in rare locations or special circumstances | 2020 | Europe, Americas | No | Other digestive diseases |
| Italian Society of Colorectal Surgery | Consensus statement of the Italian society of colorectal surgery (SICCR): management and treatment of hemorrhoidal disease | 2020 | Italy | No | Digestive diseases |
| **Endocrine/Blood/Immune disorders** | | | | | |
| American Thyroid Association | Guidelines of the American Thyroid Association for the diagnosis and management of thyroid disease during pregnancy and postpartum | 2011 | United States of America | Yes | Disorders of thyroid gland |
| American Heart Association / American Stroke Association | Diagnosis and management of cerebral venous thrombosis: a statement for healthcare professionals from the American Heart Association/American Stroke Association | 2011 | United States of America | No | Cerebral venous thrombosis |
| Endocrine Society | Clinical practice guideline for the diagnosis and treatment of hyperprolactinemia | 2011 | International | No | Disorders of other endocrine glands |
| Endocrine Society | Diagnosis and treatment of hyperprolactinemia: an Endocrine Society clinical practice guideline | 2011 | International | No | Disorders of other endocrine glands |
| Italian Thyroid Association /Italian Association of Clinical Endocrinologists | Hyperthyroidism and pregnancy. An Italian Thyroid Association (AIT) and Italian Association of Clinical Endocrinologists (AME) joint statement for clinical practice | 2011 | Italy | Yes | Disorders of thyroid gland |
| Polish Society of Endocrinology | Management of thyroid diseases during pregnancy | 2011 | Poland | Yes | Disorders of thyroid gland |
| Expert Panel on FH National Lipid Association Expert Panel on Familial Hypercholesterolemia | Familial hypercholesterolemia: screening, diagnosis and management of pediatric and adult patients: clinical guidance from the National Lipid Association Expert Panel on Familial Hypercholesterolemia | 2011 | United States of America | No | Metabolic disorders |
| British Society of Gastroenterology | British Society of Gastroenterology (BSG) guidelines for management of autoimmune hepatitis | 2011 | United Kingdom | No | Autoimmune hepatitis |
| American Thyroid Association / American Association of Clinical Endocrinologists | Hyperthyroidism and other causes of thyrotoxicosis: management guidelines of the American Thyroid Association and American Association of Clinical Endocrinologists | 2011 | United States of America | No | Disorders of thyroid gland |
| Spanish Study Group on Bradykinin-Induced Angioedema | Consensus statement on the diagnosis, management, and treatment of angioedema mediated by bradykinin. Part II. Treatment, follow-up, and special situations | 2011 | Spain | No | Other |
| Royal College of Obstetricians and Gynecologists | Management of Sickle Cell Disease in Pregnancy | 2011 | United Kingdom | Yes | Sickle cell disorder |
| The Society of Nuclear Medicine and Molecular Imaging | The SNMMI practice guideline for therapy of thyroid disease with 131I 3.0 | 2012 | United States of America | No | Disorders of thyroid gland |
| American Association of Clinical Endocrinologists / American Thyroid Association | Clinical practice guidelines for hypothyroidism in adults: cosponsored by the American Association of Clinical Endocrinologists and the American Thyroid Association | 2012 | United States of America | No | Disorders of thyroid gland |
| World Allergy Organization | WAO Guideline for the Management of Hereditary Angioedema | 2012 | International | No | Other congenital anomalies |
| Endocrine Society | Management of thyroid dysfunction during pregnancy and postpartum: an Endocrine Society clinical practice guideline | 2012 | International | Yes | Disorders of thyroid gland |
| International consensus | International consensus and practical guidelines on the gynecologic and obstetric management of female patients with hereditary angioedema caused by C1 inhibitor deficiency | 2012 | International | Yes | Diseases of the blood and blood-forming organs and certain disorders involving the immune mechanism |
| Dutch College of General Practitioners | Zwangerschap en kraamperiode (M32) | 2012 | Netherlands | Yes | Disorders of thyroid gland |
| Brazilian Society of Rheumatology | Diretrizes para o tratamento da síndrome do anticorpo antifosfolipídeo | 2013 | Brazil | No | Diseases of the blood and blood-forming organs and certain disorders involving the immune mechanism |
| Dutch College of General Practitioners | Summary of the 'Thyroid disorders' guideline of the Dutch College of General Practitioners' (NHG) | 2013 | Netherlands | No | Disorders of thyroid gland |
| Brazilian Society of Endocrinology and Metabolism | The Brazilian consensus for the diagnosis and treatment of hyperthyroidism: recommendations by the Thyroid Department of the Brazilian Society of Endocrinology and Metabolism | 2013 | Brazil | No | Disorders of thyroid gland |
| European Society of Endocrinology | A consensus on the diagnosis and treatment of acromegaly complications | 2013 | Europe | No | Disorders of other endocrine glands |
| Neuroendocrinology Group of the SEEN | Clinical guidelines for diagnosis and treatment of prolactinoma and hyperprolactinemia | 2013 | Spain | No | Disorders of other endocrine glands |
| Latin American Thyroid Society | Clinical practice guidelines for the management of hypothyroidism | 2013 | Brazil | No | Disorders of thyroid gland |
| Brazilian Society of Endocrinology and Metabolism | Consenso brasileiro para a abordagem clínica e tratamento do hipotireoidismo subclínico em adultos: recomendações do Departamento de Tireoide da Sociedade Brasileira de Endocrinologia e Metabologia | 2013 | Brazil | No | Disorders of thyroid gland |
| Brazilian consensus | Thyroid nodules and differentiated thyroid cancer: update on the Brazilian consensus | 2013 | Brazil | No | Disorders of thyroid gland |
| Expert Consensus | Consensus statement on the diagnosis, treatment and follow-up of patients with primary adrenal insufficiency | 2014 | Europe | No | Disorders of other endocrine glands |
| European Society of Endocrinology | Acromegaly: an endocrine society clinical practice guideline | 2014 | Europe | No | Disorders of other endocrine glands |
| Royal College of Obstetricians and Gynecologists | Management of Beta Thalassaemia in Pregnancy | 2014 | United Kingdom | No | Haemoglobinopathies/hemolytic anemias |
| The Swedish Society Obstetrics Gynecology | Thyroid Disease in the Perinatal Period | 2014 | Sweden | Yes | Disorders of thyroid gland |
| The Sociedad Andaluza de Endocrinología y Nutrición | Thyroid dysfunction in pregnancy. Consensus document. Andalusian Society of Endocrinology and Nutrition (SAEN) | 2015 | Spain | Yes | Disorders of thyroid gland |
| Ministry of Health, Labor and Welfare | Consensus report for the management of pregnancy with primary immune thrombocytopenia | 2015 | Japan | Yes | Disorders of thyroid gland |
| Expert consensus | Hereditary angioedema treatments: Recommendations from the French national centre for angioedema (Bordeaux consensus 2014) | 2015 | France | No | Other congenital anomalies |
| Japan Endocrine Society | Diagnosis and treatment of adrenal insufficiency including adrenal crisis: a Japan Endocrine Society clinical practice guideline [Opinion] | 2016 | Japan | No | Other disorders of adrenal gland |
| American Thyroid Association | 2016 American Thyroid Association Guidelines for Diagnosis and Management of Hyperthyroidism and Other Causes of Thyrotoxicosis | 2016 | United States of America | No | Disorders of thyroid gland |
| British Committee for Standards in Haematology | Guidelines for the diagnosis and management of adult aplastic anaemia | 2016 | United Kingdom | No | Haemoglobinopathies/hemolytic anemias |
| Italian Association of Clinical Endocrinologists | Replacement therapy for primary hypothyroidism: a brief guide for clinical practice | 2016 | Italy | No | Disorders of thyroid gland |
| American Association of Clinical Endocrinologists/American College of Endocrinology/Italian Association of Clinical Endocrinologists | American Association of Clinical Endocrinologists, American College of Endocrinology, Associazione Medici Endocrinologi Medical Guidelines for Clinical Practice for the Diagnosis and Management of Thyroid Nodules--2016 Update | 2016 | United States of America | No | Disorders of thyroid gland |
| Endocrine Society | Hormonal Replacement in Hypopituitarism in Adults: An Endocrine Society Clinical Practice Guideline | 2016 | International | No | Disorders of other endocrine glands |
| Endocrine Society | Diagnosis and Treatment of Primary Adrenal Insufficiency: An Endocrine Society Clinical Practice Guideline | 2016 | International | No | Disorders of other endocrine glands |
| American College of Obstetricians and Gynecologists | Practice Bulletin No. 166: Thrombocytopenia in Pregnancy | 2016 | United States of America | Yes | Diseases of the blood and blood-forming organs and certain disorders involving the immune mechanism |
| British Society for Haematology | Red cell transfusion in sickle cell disease Part II | 2016 | United Kingdom | No | Haemoglobinopathies/hemolytic anemias |
| Ministry of Health | Ministry of Health Clinical Practice Guidelines: Lipids | 2017 | Singapore | No | Metabolic disorders |
| Japan College of Rheumatology | Clinical practice guideline for Sjögren's syndrome 2017 | 2017 | Japan | No | Other musculoskeletal disorders |
| British Society for Haematology | The British Society for Rheumatology guideline for the management of adults with primary Sjögren's Syndrome | 2017 | United Kingdom | No | Other musculoskeletal disorders |
| European Stroke Organization/ European Academy of Neurology | European Stroke Organization guideline for the diagnosis and treatment of cerebral venous thrombosis - endorsed by the European Academy of Neurology | 2017 | Europe | No | Other |
| American Thyroid Association | 2017 Guidelines of the American Thyroid Association for the Diagnosis and Management of Thyroid Disease During Pregnancy and the Postpartum | 2017 | United States of America | Yes | Disorders of thyroid gland |
| The Korean Endocrine Society /Korean Adrenal Gland and Endocrine Hypertension Study Group | Guidelines for the Management of Adrenal Incidentaloma: the Korean Endocrine Society, Committee of Clinical Practice Guidelines | 2017 | South Korea | No | Other disorders of adrenal gland |
| American College of Cardiology | 2017 Focused update of the 2016 ACC Expert consensus decision pathway on the role of non-statin therapies for LDL-cholesterol lowering in the management of atherosclerotic cardiovascular disease risk | 2017 | United States of America | No | Metabolic disorders |
| National Institute for Health and Care Excellence | Cystic fibrosis: diagnosis and management | 2017 | United Kingdom | No | Metabolic disorders |
| Canadian Cardiovascular Society | Canadian Cardiovascular Society Position Statement on Familial Hypercholesterolemia: Update 2018 | 2018 | Canada | No | Metabolic disorders |
| Melbourne public hospitals consensus | Subclinical hypothyroidism during pregnancy: the Melbourne public hospitals consensus | 2018 | Australia | Yes | Disorders of thyroid gland |
| National Health and Medical Research Council | Translation and implementation of the Australian-led PCOS guideline: clinical summary and translation resources from the International Evidence-based Guideline for the Assessment and Management of Polycystic Ovary Syndrome | 2018 | Australia | No | Disorders of other endocrine glands |
| Expert consensus | Treatment of adult Graves' disease | 2018 | France | No | Disorders of thyroid gland |
| Society of Obstetricians and Gynaecologists of Canada | No. 163-Gynaecological and Obstetric Management of Women With Inherited Bleeding Disorders | 2018 | United States of America | Yes | Diseases of the blood and blood-forming organs and certain disorders involving the immune mechanism |
| American College of Obstetricians and Gynecologists | ACOG Practice Bulletin No. 197: Inherited Thrombophilias in Pregnancy | 2018 | United States of America | Yes | Diseases of the blood and blood-forming organs and certain disorders involving the immune mechanism |
| Royal Australian and New Zealand College of Obstetricians and Gynaecologists | Subclinical hypothyroidism and hypothyroidism in pregnancy | 2018 | Australia, New Zealand | Yes | Disorders of thyroid gland |
| European reference network for rare vascular diseases | European reference network for rare vascular diseases (VASCERN) consensus statement for the screening and management of patients with pathogenic ACTA2 variants | 2019 | Europe | No | Diseases of the blood and blood-forming organs and certain disorders involving the immune mechanism |
| European Alliance of Associations for Rheumatology | EULAR recommendations for the management of antiphospholipid syndrome in adults | 2019 | Europe | No | Diseases of the blood and blood-forming organs and certain disorders involving the immune mechanism |
| International Group of Experts | Updated international consensus report on the investigation and management of primary immune thrombocytopenia | 2019 | United States of America | No | Diseases of the blood and blood-forming organs and certain disorders involving the immune mechanism |
| Expert consensus | Consensus on the investigation of thrombophilia in women and clinical management | 2019 | Brazil | No | Diseases of the blood and blood-forming organs and certain disorders involving the immune mechanism |
| Expert consensus | Diagnosing and treating antiphospholipid syndrome: a consensus paper | 2019 | Netherlands | No | Diseases of the blood and blood-forming organs and certain disorders involving the immune mechanism |
| Australian Hemophilia Centre Directors' Organization | Updated Australian consensus statement on management of inherited bleeding disorders in pregnancy | 2019 | Australia | Yes | Diseases of the blood and blood-forming organs and certain disorders involving the immune mechanism |
| American College of Obstetricians and Gynecologists | ACOG Practice Bulletin No. 207: Thrombocytopenia in Pregnancy | 2019 | United States of America | Yes | Diseases of the blood and blood-forming organs and certain disorders involving the immune mechanism |
| National Institute for Health and Care Excellence | Hyperparathyroidism (primary): diagnosis, assessment and initial management | 2019 | United Kingdom | No | Disorders of thyroid gland |
| National Health and Medical Research Council | Pregnancy Care: Clinical Practice Guidelines | 2020 | Australia | Yes | Disorders of thyroid gland |
| International Society of Thrombosis and Hemostasis | ISTH guidelines for treatment of thrombotic thrombocytopenic purpura | 2020 | International | No | Diseases of the blood and blood-forming organs and certain disorders involving the immune mechanism |
| European Alliance of Associations for Rheumatology | EULAR recommendations for a core data set for pregnancy registries in rheumatology | 2020 | Europe | Yes | Rheumatoid arthritis |
| American Society of Hematology | American Society of Hematology 2020 guidelines for sickle cell disease: transfusion support | 2020 | United States of America | No | Sickle cell disorder |
| American College of Obstetricians and Gynecologists | Thyroid Disease in Pregnancy: ACOG Practice Bulletin, Number 223 | 2020 | United States of America | Yes | Disorders of thyroid gland |
| American Society of Hematology/ International Society of Thrombosis and Hemostasis / National Hemophilia Foundation / World Federation of Hemophilia | ASH ISTH NHF WFH 2021 guidelines on the management of von Willebrand disease | 2021 | International | No | Diseases of the blood and blood-forming organs and certain disorders involving the immune mechanism |
| Association of Anesthetists | Guideline on the peri-operative management of patients with sickle cell disease | 2021 | United Kingdom | No | Sickle cell disorder |
| **Genitourinary diseases** | | | | | |
| European Society of Urogenital Radiology | Acute abdominal and pelvic pain in pregnancy: ESUR recommendations | 2013 | Europe | Yes | Gynecological diseases; Urolithiasis |
| Society of Obstetricians and Gynaecologists of Canada | The management of uterine leiomyomas | 2015 | Canada | No | Gynecological diseases |
| Kidney and Pregnancy Study Group of Italian Society of Nephrology | Best practices on pregnancy on dialysis: the Italian Study Group on Kidney and Pregnancy | 2015 | Italy | Yes | Kidney diseases |
| American College of Radiology | Acute Onset Flank Pain - Suspicion of Stone Disease (Urolithiasis) | 2015 | United States of America | No | Urolithiasis |
| European Academy of Dermatology and Venereology | 2016 European guideline for the management of vulval conditions | 2016 | Europe | No | Gynecological diseases |
| European Association of Urology | EAU Guidelines on Interventional Treatment for Urolithiasis | 2016 | Europe | No | Urolithiasis |
| American Urological Association Working Group/Endourological Society | Surgical management of stones: American Urological Association/Endourological Society Guideline, PART I | 2016 | United States of America | No | Urolithiasis; Kidney diseases |
| Association of British Clinical Diabetologists | Association of British Clinical Diabetologists - Renal Association (ABCD-RA) Clinical Practice Guidelines for Management of Lipids in Adults with Diabetes Mellitus and Nephropathy and/or Chronic Kidney Disease | 2017 | United Kingdom | No | Kidney diseases |
| European Renal Association–European Dialysis and Transplant Association and European Society for Paediatric Nephrology/ the German Society of Obstetrics and Gynecology/German Society of Perinatal Medicine, German Society of Ultrasound in Medicine/PKD International | Perinatal diagnosis, management, and follow-up of cystic renal diseases | 2018 | Europe | Yes | Genitourinary diseases |
| The Renal Foundation | Clinical practice guideline on pregnancy and renal disease | 2019 | United Kingdom | Yes | Kidney diseases |
| Renal Association | Renal Association Clinical Practice Guideline on Haemodialysis | 2019 | United Kingdom | No | Kidney diseases |
| European Association of Urology | Urolithiasis | 2019 | Europe | No | Urolithiasis |
| Danish Society of Obstetrics and Gynecology | Urological diseases and pregnancy | 2019 | Denmark | Yes | Kidney diseases |
| Danish Society of Obstetrics and Gynecology | Chronic renal diseases and pregnancy | 2019 | Denmark | Yes | Kidney diseases |
| National Institute for Health and Care Excellence | Renal and ureteric stones: assessment and management | 2019 | United Kingdom | No | Kidney diseases |
| **Mental and substance use disorders** | | | | | |
| Ministerio de Salud | Intervenciones breves para reducir el consumo de alcohol de riesgo: guía técnica para atención primaria en salud | 2011 | Chile | No | Alcohol use disorders |
| National Health and Medical Research Council | Consensus-Based Clinical Practice Guideline for the Management of Volatile Substance Use in Australia | 2011 | Australia | No | Drug use disorders |
| beyondblue | Clinical practice guidelines for depression and related disorders - anxiety, bipolar disorder and puerperal psychosis - in the perinatal period. A guideline for primary care health professionals (February 2011) | 2011 | Australia | Yes | Anxiety disorders; Bipolar disorder; Depressive disorders |
| British Association for Psychopharmacology | BAP updated guidelines: evidence-based guidelines for the pharmacological management of substance abuse, harmful use, addiction and comorbidity: recommendations from BAP | 2012 | United Kingdom | No | Drug use disorders |
| American College of Obstetricians and Gynecologists | Committee Opinion No. 711: Opioid Use and Opioid Use Disorder in Pregnancy | 2012 | United States of America | Yes | Drug use disorders |
| British Association for Psychopharmacology | Evidence based guidelines for the pharmacological management of substance abuse, harmful use, addiction and comorbidity | 2012 | United Kingdom | No | Alcohol use disorders; Other mental and behavioral disorders |
| Scottish Intercollegiate Guidelines Network | SIGN 127: Management of perinatal mood disorders | 2012 | Scotland | Yes | Other mental and behavioral disorders |
| Guideline Expert Advisory Committee | Detection and management of mood disorders in the maternity setting: the Australian Clinical Practice Guidelines | 2013 | Australia | Yes | Depressive disorders |
| Scottish Intercollegiate Guidelines Network | Management of schizophrenia | 2013 | Scotland | No | Schizophrenia |
| Government of South Australia | Substance use in pregnancy | 2013 | Australia | Yes | Alcohol use disorders; Drug use disorders |
| National Institute for Health and Care Excellence | Antenatal and postnatal mental health: clinical management and service guidance | 2014 | United Kingdom | Yes | General mental disorders |
| Expert consensus | Canadian clinical practice guidelines for the management of anxiety, posttraumatic stress and obsessive-compulsive disorders | 2014 | Canada | No | Anxiety disorders |
| BC Reproductive Mental Health Program | Best Practice Guidelines for Mental Health Disorders in the Perinatal Period | 2014 | Canada | Yes | Anxiety disorders; Bipolar disorder; Depressive disorders; Other mental and behavioral disorders |
| World Health Organization | Guidelines for identification and management of substance use and substance use disorders in pregnancy | 2014 | International | Yes | Drug use disorders |
| NSW Ministry of Health | Clinical Guidelines for the Management of Substance Use During Pregnancy, Birth and the Postnatal Period | 2014 | Australia | Yes | Drug use disorders |
| U.S. Preventive Services Task Force | Behavioral and Pharmacotherapy Interventions for Tobacco Smoking Cessation in Adults, Including Pregnant Women: US Preventive Services Task Force Recommendation Statement | 2015 | United States of America | No | Drug use disorders |
| French Alcohol Society/ European Federation of Addiction Societies | Pharmacotherapy for Alcohol Dependence: The 2015 Recommendations of the French Alcohol Society, Issued in Partnership with the European Federation of Addiction Societies | 2015 | France | No | Alcohol use disorders |
| Ministry of Health | Ministry of Health Clinical Practice Guidelines: Anxiety Disorders | 2015 | Singapore | No | Anxiety disorders |
| American Society of Addiction Medicine | American Society of Addiction Medicine (ASAM) National Practice Guideline for the Use of Medications in the Treatment of Addiction Involving Opioid Use | 2015 | United States of America | No | Drug use disorders |
| World Federation of Societies of Biological Psychiatry | World Federation of Societies of Biological Psychiatry (WFSBP) Guidelines for Biological Treatment of Schizophrenia. Part 3: Update 2015 Management of special circumstances: Depression, Suicidality, substance use disorders and pregnancy and lactation | 2015 | International | Yes | Depressive disorders |
| Ministry of Health | Clinical Practice Guidelines on Anxiety Disorders | 2015 | Singapore | No | Anxiety disorders |
| Royal Australian and New Zealand College of Psychiatrists | Guidance on the clinical management of depressive and bipolar disorders, specifically focusing on diagnosis and treatment strategies | 2015 | Australia, New Zealand | No | Bipolar disorder; Depressive disorders |
| World Health Organization | Pregnancy, childbirth, postpartum and newborn care | 2015 | International | Yes | Depressive disorders |
| Canadian Network for Mood and Anxiety Treatments | Canadian Network for Mood and Anxiety Treatments (CANMAT) 2016 Clinical Guidelines for the Management of Adults with Major Depressive Disorder: Section 6. Special Populations: Youth, Women, and the Elderly | 2016 | Canada | No | Depressive disorders |
| U.S. Preventive Services Task Force | Screening for Depression in Adults: US Preventive Services Task Force Recommendation Statement | 2016 | United States of America | No | Depressive disorders |
| Government of South Australia | Anxiety and depression in the perinatal period | 2016 | Australia | Yes | Anxiety disorders; Depressive disorders |
| British Association for Psychopharmacology | Evidence-based Guidelines for Treating Bipolar Disorder | 2016 | United Kingdom | No | Bipolar disorder |
| Royal Australian and New Zealand College of Psychiatrists | Guidance addressing all aspects of the care of people with schizophrenia and related disorders. Includes correct diagnosis, symptom relief and recovery of social function | 2016 | Australia, New Zealand | No | Schizophrenia |
| Royal Australian and New Zealand College of Psychiatrists | Royal Australian and New Zealand College of Psychiatrists clinical practice guidelines for the management of schizophrenia and related disorders | 2016 | Australia, New Zealand | No | Schizophrenia |
| Society of Obstetricians and Gynaecologists of Canada | No. 349-Substance Use in Pregnancy | 2017 | Canada | Yes | Drug use disorders |
| Expert consensus | Recommendations for buprenorphine and methadone therapy in opioid use disorder: a European consensus | 2017 | Europe | No | Drug use disorders |
| National Institute for Health and Care Excellence | Eating disorders: recognition and treatment | 2017 | United Kingdom | No | Eating disorders |
| American Psychiatric Association | Pharmacological Treatment of Patients with Alcohol Use Disorder | 2017 | United States of America | No | Alcohol use disorders |
| U.S. Preventive Services Task Force | Screening and Behavioral Counseling Interventions to Reduce Unhealthy Alcohol Use in Adolescents and Adults: US Preventive Services Task Force Recommendation Statement | 2018 | United States of America | No | Alcohol use disorders |
| Korean Society for Affective Disorders | Korean Medication Algorithm for Depressive Disorders 2017: Third Revision | 2018 | South Korea | No | Other mental and behavioral disorders |
| American College of Obstetricians and Gynecologists | Screening for Perinatal Depression | 2018 | United States of America | Yes | Depressive disorders |
| Registered Nurses' Association of Ontario | Assessment and Interventions for Perinatal Depression | 2018 | Canada | Yes | Depressive disorders |
| Royal Australian and New Zealand College of Psychiatrists | Guidance on the clinical management of anxiety disorders, specifically focusing on diagnosis and treatment strategies | 2018 | Australia, New Zealand | No | Anxiety disorders |
| Royal Australian and New Zealand College of Obstetricians and Gynaecologists | Substance use in pregnancy | 2018 | Australia, New Zealand | Yes | Drug use disorders |
| Royal Australian and New Zealand College of Psychiatrists | Royal Australian and New Zealand College of Psychiatrists clinical practice guidelines for the treatment of panic disorder, social anxiety disorder and generalised anxiety disorder | 2018 | Australia, New Zealand | No | Anxiety disorders |
| Polish Psychiatric Association | Recommendations of the Polish Psychiatric Association regarding the treatment of affective disorders in women of childbearing age. Part II: Bipolar disorder | 2019 | Poland | No | Bipolar disorder |
| World Federation of Societies of Biological Psychiatry / International Association for Women's Mental Health | WFSBP and IAWMH Guidelines for the treatment of alcohol use disorders in pregnant women | 2019 | International | Yes | Alcohol use disorders |
| U.S. Preventive Services Task Force | Perinatal Depression: Preventive Interventions | 2019 | United States of America | Yes | Depressive disorders |
| Ministry of Health | Management of Major Depressive Disorder (2nd Edition) | 2019 | Malaysia | No | Depressive disorders |
| Women's Preventive Services Initiative | Screening for Anxiety in Adolescent and Adult Women: A Recommendation From the Women's Preventive Services Initiative | 2020 | United States of America | No | Anxiety disorders |
| American Society of Addiction Medicine | The ASAM Clinical Practice Guideline on Alcohol Withdrawal Management | 2020 | United States of America | No | Alcohol use disorders |
| Society of Obstetricians and Gynaecologists of Canada | Screening and Counselling for Alcohol Consumption During Pregnancy | 2020 | Canada | Yes | Alcohol use disorders |
| U.S. Preventive Services Task Force | Unhealthy Drug Use: Screening | 2020 | United States of America | No | Drug use disorders |
| Government of South Australia | Eating Disorders and Pregnancy | 2020 | Australia | No | Eating disorders |
| British Columbia Centre on Substance Use | Provincial Guideline for the Clinical Management of High Risk Drinking and Alcohol Use Disorder: Pregnancy Supplement | 2020 | Canada | Yes | Alcohol use disorders |
| US Preventive Services Task Force | Interventions for Tobacco Smoking Cessation in Adults, Including Pregnant Persons: US Preventive Services Task Force | 2021 | United States of America | No | Drug use disorders |
| American Psychiatric Association | Practice Guideline for the treatment of patients with schizophrenia. Third edition | 2021 | United States of America | No | Schizophrenia |
| Royal Australian and New Zealand College of Psychiatrists | The 2020 Royal Australian and New Zealand College of Psychiatrists clinical practice guidelines for mood disorders | 2021 | Australia, New Zealand | No | Anxiety disorders; Bipolar disorder; Depressive disorders |
| **Multiple categories** | | | | | |
| Japan Society of Obstetrics and Gynecology / Japan Association of Obstetricians and Gynecologists | Guidelines for obstetrical practice in Japan: Japan Society of Obstetrics and Gynecology (JSOG) and Japan Association of Obstetricians and Gynecologists (JAOG) 2011 edition | 2011 | Japan | Yes | Diabetes-Gestational; Disorders of thyroid gland |
| Society of Obstetricians and Gynaecologists of Canada | Adolescent Pregnancy Guidelines | 2015 | Canada | Yes | Disorders of thyroid gland; Other mental and behavioral disorders |
| European Alliance of Associations for Rheumatology | EULAR recommendations for women's health and the management of family planning, assisted reproduction, pregnancy and menopause in patients with systemic lupus erythematosus and/or antiphospholipid syndrome | 2016 | Europe | Yes | Diseases of the blood and blood-forming organs and certain disorders involving the immune mechanism; Other musculoskeletal disorders |
| National Institute for Health and Care Excellence | Intrapartum care for women with existing medical conditions or obstetric complications and their babies | 2019 | United Kingdom | Yes | General cardiovascular diseases; Asthma |
| **Musculoskeletal diseases** | | | | | |
| British Society for Haematology | Guidelines on the diagnosis and management of thrombotic thrombocytopenic purpura and other thrombotic microangiopathies | 2012 | United Kingdom | Yes | Other musculoskeletal disorders |
| Joint European League Against Rheumatism and European Renal Association-European Dialysis and Transplant Association | Joint European League Against Rheumatism and European Renal Association-European Dialysis and Transplant Association (EULAR/ERA-EDTA) recommendations for the management of adult and paediatric lupus nephritis | 2012 | Europe | No | Other musculoskeletal disorders |
| British Society of Rheumatology | Guideline for the use of intravenous tocilizumab in the treatment of adult patients with rheumatoid arthritis | 2013 | United Kingdom | No | Rheumatoid arthritis |
| Mexican College of Rheumatology | Clinical practice guidelines for the management of pregnancy in women with autoimmune rheumatic diseases of the Mexican College of Rheumatology. Part II | 2015 | Mexico | Yes | Rheumatoid arthritis |
| Task Group of South African Rheumatologists | The specialist physician's approach to rheumatoid arthritis in South Africa | 2016 | South Africa | No | Rheumatoid arthritis |
| Thai Rheumatism Association | 2016 updated Thai Rheumatism Association Recommendations for the use of biologic and targeted synthetic disease-modifying anti-rheumatic drugs in patients with rheumatoid arthritis | 2017 | Thailand | No | Rheumatoid arthritis |
| The Costa Rican Association of Rheumatology | Guías de manejo de artritis reumatoide Consenso 2016 Asociación Costarricense de Reumatología | 2017 | Costa Rica | No | Rheumatoid arthritis |
| British Society of Rheumatology | BSR guideline on the management of gout | 2017 | United Kingdom | No | Gout |
| Canadian Rheumatology Association | Canadian Rheumatology Association Recommendations for the Assessment and Monitoring of Systemic Lupus Erythematosus | 2018 | Canada | No | Other musculoskeletal disorders |
| Korean College of Rheumatology | Korean Guideline for the Prevention and Treatment of Glucocorticoid-induced Osteoporosis | 2018 | Korea | No | Other musculoskeletal disorders |
| The Asia-Pacific League of Associations for Rheumatology | 2018 update of the APLAR recommendations for treatment of rheumatoid arthritis | 2019 | Asia-Pacific | No | Rheumatoid arthritis |
| Kuwait Association of Rheumatology | Kuwait association of rheumatology 2018 treatment recommendations for patients with rheumatoid arthritis | 2019 | Kuwait | No | Rheumatoid arthritis |
| Joint European League Against Rheumatism and European Renal Association-European Dialysis and Transplant Association | 2019 Update of the Joint European League Against Rheumatism and European Renal Association-European Dialysis and Transplant Association (EULAR/ERA-EDTA) recommendations for the management of lupus nephritis | 2020 | Europe | No | Other musculoskeletal disorders |
| American College of Radiology | 2020 American College of Rheumatology Guideline for the Management of Reproductive Health in Rheumatic and Musculoskeletal Diseases | 2020 | United States of America | No | General musculoskeletal disease |
| American College of Radiology | American College of Rheumatology Guideline for the Management of Reproductive Health in Rheumatic and Musculoskeletal Diseases | 2020 | United States of America | Yes | General musculoskeletal disease |
| **Neurological Conditions** | | | | | |
| Canadian Headache Society | Canadian Headache Society guideline for migraine prophylaxis | 2012 | Canada | No | Migraine |
| Societe francaise d'etude des migraines et des cephalees | Guidelines for the diagnosis and management of migraine in adults and children | 2012 | France | No | Migraine |
| Expert consensus | Recommendations for the treatment of epilepsy in adult patients in general practice in Belgium: an update | 2012 | Belgium | No | Epilepsy |
| Danish Headache Society | Reference programme: diagnosis and treatment of headache disorders and facial pain. Danish Headache Society, 2nd Edition, 2012 | 2012 | Denmark | No | Non-migraine headache |
| National Institute for Health and Care Excellence | Epilepsies: diagnosis and management | 2012 | United Kingdom | No | Epilepsy |
| National Institute for Health and Care Excellence | Headaches in over 12s: diagnosis and management | 2012 | United Kingdom | No | Migraine |
| Canadian Headache Society | Canadian Headache Society Guideline: acute drug therapy for migraine headache | 2013 | Canada | No | Migraine |
| National Institute for Health and Care Excellence | Diagnosis and management of headaches in young people and adults: NICE guideline | 2013 | United Kingdom | No | Non-migraine headache |
| International Restless Legs Syndrome Study Group | Consensus clinical practice guidelines for the diagnosis and treatment of restless legs syndrome/Willis-Ekbom disease during pregnancy and lactation | 2015 | International | Yes | Other neurological conditions |
| The Spanish Society of Neurology | The Spanish Society of Neurology's official clinical practice guidelines for epilepsy. Special considerations in epilepsy: comorbidities, women of childbearing age, and elderly patients | 2015 | Spain | No | Epilepsy |
| Ministry of Health | Management of Multiple Sclerosis | 2015 | Malaysia | No | Multiple sclerosis |
| Association of British Neurologists | Guidelines for prescribing disease-modifying treatments in multiple sclerosis | 2015 | United Kingdom | No | Multiple sclerosis |
| American Academy of Neurology | Appendix B: AAN Summary of Evidence-Based Guideline for Clinicians: Management Issues for Women With Epilepsy--Focus on Pregnancy: Obstetrical Complications and Change in Seizure Frequency | 2016 | United States of America | Yes | Epilepsy |
| Royal College of Obstetricians and Gynecologists | Epilepsy in Pregnancy | 2016 | United Kingdom | Yes | Epilepsy |
| Toward Optimized Practice | Primary Care Management of Headache in Adults Clinical Practice Guideline | 2016 | Canada | No | Migraine; Non-migraine headache |
| Polish Society of Epileptology/Polish Gynecological Society | Managing epilepsy in women of childbearing age - Polish Society of Epileptology and Polish Gynecological Society Guidelines | 2017 | Poland | No | Epilepsy |
| Taiwan Headache Society | Medical Treatment Guidelines for Preventive Treatment of Migraines | 2017 | Taiwan | No | Migraine |
| Taiwan Headache Society | Medical Treatment Guidelines for Acute Migraine Attacks | 2017 | Taiwan | No | Migraine |
| Antiepileptic Drug Management in Pregnancy | Development of a core outcome set for epilepsy in pregnancy (E-CORE): a national multi-stakeholder modified Delphi consensus study | 2017 | United Kingdom | Yes | Epilepsy |
| SA Maternal & Neonatal Clinical Network | Seizures in pregnancy | 2017 | Australia | Yes | Epilepsy |
| European Academy of Neurology / European Committee of Treatment of Research in Multiple Sclerosis | ECTRIMS/EAN Guideline on the pharmacological treatment of people with multiple sclerosis | 2018 | Europe | No | Multiple sclerosis |
| Scottish Intercollegiate Guidelines Network | Pharmacological management of migraine | 2018 | Scotland | No | Migraine |
| Scottish Intercollegiate Guidelines Network | Diagnosis and management of epilepsy in adults | 2018 | Scotland | No | Epilepsy |
| Government of South Australia | Epilepsy and pregnancy management | 2018 | Australia | Yes | Epilepsy |
| American Academy of Neurology | Practice Guideline Recommendations Summary: Disease-modifying Therapies for Adults with Multiple Sclerosis | 2018 | United States of America | No | Multiple sclerosis |
| American College of Radiology | ACR Appropriateness Criteria Headache | 2019 | United States of America | No | Migraine |
| International League Against Epilepsy Task Force | Management of epilepsy in pregnancy: a report from the International League Against Epilepsy Task Force on Women and Pregnancy | 2019 | Multinational | Yes | Epilepsy |
| Brazilian Headache Society | Consensus of the Brazilian Headache Society on the treatment of chronic migraine | 2019 | Brazil | No | Migraine |
| Association of British Neurologists | UK consensus on pregnancy in multiple sclerosis: 'Association of British Neurologists' guidelines | 2019 | United Kingdom | Yes | Multiple sclerosis |
| Colombia Neurology Association | Tratamiento del estado epiléptico, consenso de expertos. Asociación Colombiana de Neurología, Comité de Epilepsia | 2019 | Colombia | No | Epilepsy |
| Working group on multiple sclerosis in Argentina | Consensus recommendations for family planning and pregnancy in multiple sclerosis in Argentina | 2020 | Argentina | Yes | Multiple sclerosis |
| The Swedish Medical Products Agency | Evidence-based anti-seizure monotherapy in newly diagnosed epilepsy: A new approach | 2020 | Sweden | No | Epilepsy |
| American College of Obstetricians and Gynecologists | Gynecologic Management of Adolescents and Young Women With Seizure Disorders | 2020 | United States of America | No | Epilepsy |
| **Sense organ diseases** | | | | | |
| Ministry of Health | Management of Glaucoma | 2017 | Malaysia | No | Glaucoma |
| **Respiratory diseases** | | | | | |
| British Thoracic Society / Scottish Intercollegiate Guidelines Network | British guideline on the management of asthma | 2014 | United Kingdom | No | Asthma |
| National Institute for Health and Care Excellence | Management of an Acute Asthma Attack in Adults (aged 16 years and older) | 2015 | Ireland | No | Asthma |
| Asthma and Respiratory Foundation NZ | Asthma and Respiratory Foundation NZ adult asthma guidelines: a quick reference guide | 2016 | New Zealand | No | Asthma |
| Government of South Australia | Asthma in pregnancy | 2016 | Australia | Yes | Asthma |
| British Society of Allergy and Clinical Immunology | BSACI guideline for the diagnosis and management of allergic and non-allergic rhinitis (Revised Edition 2017; First edition 2007) | 2017 | United Kingdom | No | Other respiratory diseases |
| Japanese Society of Allergology | Japanese guidelines for allergic rhinitis 2017 | 2017 | Japan | No | Other respiratory diseases |
| La Sociedad Mexicana de Neumología y Cirugía de Tórax | Mexican Asthma Guidelines: GUIMA 2017 | 2017 | Mexico | No | Asthma |
| Korean Medical Association | Clinical diagnostic guidelines of allergic rhinitis: comprehensive treatment and consideration of special circumstances | 2017 | South Korea | No | Other respiratory diseases |
| British Thoracic Society / Scottish Intercollegiate Guidelines Network | BTS/SIGN British Guideline on the Management of Asthma | 2019 | United Kingdom | No | Asthma |
| American Academy of Allergy, Asthma, and Immunology/ American College of Asthma, Allergy and Immunology | Rhinitis 2020: A practice parameter update | 2020 | United States of America | No | Other respiratory diseases |
| Japanese Society of Allergology | Japanese guidelines for adult asthma 2020 | 2020 | Japan | No | Asthma |
| Saudi Thoracic Society | The Saudi Initiative for Asthma - 2021 Update: Guidelines for the diagnosis and management of asthma in adults and children | 2021 | Saudi Arabia | No | Asthma |
| **Oral conditions** | | | | | |
| European Federation of Periodontology / American Academy of Periodontology | Periodontitis and adverse pregnancy outcomes: consensus report of the Joint EFP/AAP Workshop on Periodontitis and Systemic Diseases | 2013 | International | Yes | Periodontal disease |
| **Skin diseases** | | | | | |
| National Psoriasis Foundation Medical Board | Consensus guidelines for the management of plaque psoriasis | 2012 | Canada | No | Papulosquamous disorders |
| Canadian Dermatology Association | A consensus on acne management focused on specific patient features | 2014 | Canada | No | Disorders of skin appendages |
| British Society of Allergy and Clinical Immunology | BSACI guideline for the management of chronic urticaria and angioedema | 2015 | United Kingdom | No | Urticaria and erythema |
| British Association of Dermatologists | British Association of Dermatologists guidelines for biologic therapy for psoriasis 2017 | 2017 | United Kingdom | No | Urticaria and erythema |
| British Association of Dermatologists | British Association of Dermatologists' guidelines for the management of pemphigus vulgaris 2017 | 2017 | United Kingdom | No | Diseases of the skin and subcutaneous tissue |
| Expert consensus | S3 Guideline for the treatment of psoriasis vulgaris, update - Short version part 2 - Special patient populations and treatment situations | 2018 | Germany | No | Papulosquamous disorders |
| British Association of Dermatologists | British Association of Dermatologists guidelines for the management of lichen sclerosus, 2018 | 2018 | United Kingdom | No | Atrophic disorders of skin |
| Korean Medical Association | Updated treatment guideline of chronic spontaneous urticaria | 2019 | South Korea | No | Urticaria and erythema |
| Canadian Dermatology Association | Management of Plaque Psoriasis With Biologic Therapies in Women of Child-Bearing Potential Consensus Paper | 2020 | Canada | No | Psoriasis |
| Royal Belgian Society of Dermatology and Venerology | Practical recommendations for systemic treatment in psoriasis according to age, pregnancy, metabolic syndrome, mental health, psoriasis subtype and treatment history (BETA-PSO: Belgian Evidence-based Treatment Advice in Psoriasis; part 1) | 2020 | Belgium | No | Papulosquamous disorders |
| British Association of Dermatologists | British Association of Dermatologists guidelines for biologic therapy for psoriasis 2020: a rapid update | 2020 | United Kingdom | No | Papulosquamous disorders |
| EuroGuiDerm | EuroGuiDerm Guideline on the systemic treatment of Psoriasis vulgaris – Part 2: specific clinical and comorbid situations | 2021 | Europe | No | Papulosquamous disorders |
| Taiwanese Dermatological Association | Taiwanese Dermatological Association consensus for the management of atopic dermatitis: A 2020 update | 2021 | Taiwan | No | Dermatitis and eczema |
