# Supplementary material for: Prevention and control of non-communicable diseases in antenatal, intrapartum, and postnatal care: a systematic scoping review of clinical practice guidelines since 2011
Source: BMC Med. 2022 Sep 20;20:305. doi: 10.1186/s12916-022-02508-9 (PMC9487084; doi:10.1186/s12916-022-02508-9)
Supplement: Supplementary file 6 — Additional file 6. Scope of recommendations for high priority conditions. Table 1. Gestational diabetes mellitus (GDM). Table 2. Diabetes mellitus (pre-existing). Table 3. Chronic hypertension. Table 4. Asthma. Table 5. Sickle cell disorder. Table 6. General mental disorders, bipolar disorder, psychotic disorders. Table 7. Depression and anxiety. Table 8. Substance use disorders. Table 9. Tobacco use. Table 10. Alcohol use disorder. [file 12916_2022_2508_MOESM6_ESM.docx]

**Additional file 6. Prevention and control of non-communicable diseases in antenatal, intrapartum, and postpartum care: a systematic scoping review of clinical practice guidelines since 2011**


**Table 1.** **Gestational diabetes mellitus (GDM)** – scope of recommendations across 19 clinical practice guidelines

|  | **Screening** | | **Clinical interventions** | | | | **Health systems** | |
| --- | --- | --- | --- | --- | --- | --- | --- | --- |
| **Antenatal** | **Approach to screening**  Universal  High risk  **Timing of screening**  **Type of tests**  OGTT  Fasting plasma glucose  HbA1c  Ketone testing  Urine testing | **n**  8  5  12  16  4  4  4  1 | **Blood glucose lowering pharmacological therapy**  Insulin therapy  Noninsulin antihyperglycemic agent therapy  **Glucose monitoring and targets**  Self-monitoring  Glycemic targets  Continuous glucose monitoring  **Obstetric care**  Fetal monitoring  Congenital malformation testing  Cardiotocography  Prematurity (tocolysis)  Sonography | **n**  15  7  11  12  5  8  1  2  2  2 | **Nutrition and weight management**  Nutrition therapy  Nutrition counselling and physical therapy  Nutritional supplements  Weight management  Physical activity  **Referral**  Registered dietician nutritionists  Obstetrician, endocrinologist  Diabetes educator  **Birth planning**  Selection of hospital or clinic  Timing of delivery | **n**  4  13  3  3  6  1  1  1  2  11 | **Multidisciplinary care**  Joint diabetes-antenatal clinic  Staffing (obstetrician, endocrinologist, ophthalmologist)  Staffing (diabetes educator, nutritionist, diabetologists)  **Auditing**  Medical records  Diabetes mellitus guidelines  **Laboratory testing**  Specimen collection, storage, handling, procedures  Equipment and instrumentation  Standards and controls | **n**  1  4  3  5  1  1  1  1 |
| **Intrapartum** | No recommendations identified | | **Birth**  Mode of delivery  Induction of labor  Insulin therapy  Blood glucose monitoring and targets | | | **n**  7  7  6  8 |  |  |
| **Postnatal** | **Postpartum screening**  Postnatal depression  Post-delivery surveillance  **Timing of screening**  **Type of tests**  OGTT  Fasting plasma glucose  HbA1c | **n**  1  3  7  5  2  1 | **Advice**  Lifestyle education and monitoring  Risk of type 2 diabetes  **Lactation**  **Glucose monitoring and targets**  Glucose monitoring  Glycemic targets | **n**  5  4  17  2  4 | **Pharmacological therapy**  Insulin  **Follow-up care**  Repeat testing  Obstetric care  **Referral**  Diabetes prevention programme | **n**  4  7  1  1 |  |  |

OGTT: oral glucose tolerance test

**Table 2. Diabetes mellitus (pre-existing)** – scope of recommendations across 4 clinical practice guidelines

|  | **Screening** | | **Clinical interventions** | | | | **Health systems** | |
| --- | --- | --- | --- | --- | --- | --- | --- | --- |
| **Antenatal** | **Type of tests**  HbA1c  Random blood glucose  Urine testing  Thyroid function  Liver and renal function  Nuchal translucency assessment  Serum screening  **Confirm gestational age**  Dating and viability ultrasound  **Repeat tests**  HbA1c  Urine testing  Blood pressure  Renal function | **n**  2  1  1  2  1  1  1  1  1  1  1  1 | **Advice/education**  Smoking cessation  Nutrition counselling  Physical activity  Blood glucose monitoring  Insulin pump therapy  Include certified diabetes educators  **Pharmacological therapy**  Insulin therapy  Noninsulin antihyperglycemic agent  Aspirin  Endocrinologist/obstetrician review  **Glucose monitoring and targets**  Self-monitoring  Glycemic targets  Continuous glucose monitoring  Ketonemia monitoring  **Obstetric care**  Ultrasound  Fetal echocardiogram  Umbilical blood artery flow | **n**  1  2  2  2  1  1  2  1  1  1  3  4  3  1  2  1  1 | **Routine care**  Frequency of appointments  Diabetic complication monitoring  Diabetologists/obstetrician care  Diabetes clinician weekly review  **Complications and high-risk group management**  Ketoacidosis  Proteinuria  Hyperglycemia  Hypertension  **Referral**  Endocrinologist, obstetrician  Clinical diabetes educator  Ophthalmologist  Nephrologist  Joint diabetes-antenatal clinic  **Birth planning**  Selection of hospital or clinic  Mode of delivery  Anesthetic assessment  Timing of delivery  Management of preterm birth  Consultation: specialist obstetrician | **n**  2  1  1  1  1  1  1  1  1  1  2  1  1  2  1  1  1  2  1 | **Policy**  Newborn hypoglycemia management  **Health workforce**  Training of Midwives: VRIII  Diabetes team daily ward round | **n**  1  1  1 |
| **Intrapartum** | No recommendations identified | | **Pharmacological therapy**  Basal insulin therapy  Insulin pump therapy  **Glucose monitoring and targets**  Blood glucose monitoring  Management of glucose control | **n**  3  1  2  1 | **Birth**  Induction of labor  General anesthesia  Intravenous fluids  Hypoglycemia management  Hyperglycemia management  **Elective cesarean section** | **n**  1  1  1  2  1  2 |  |  |
| **Postnatal** | **Types of tests**  Placenta histopathological examination  **Postpartum screening**  Post-delivery surveillance  Thyroiditis | **n**  1  1  1 | **Advice/education**  Contraception  Nutrition and diet  **Pharmacological therapy**  Medication review  Insulin therapy indications  Noninsulin antihyperglycemic agent therapy  **Glucose monitoring and targets**  Self-monitoring | **n**  2  2  2  3  2  2 | **Newborn care**  Blood glucose testing  Echocardiogram  Assessment for NICU  Feeding  Management of hypoglycemia  **Breastfeeding**  Medication review  Nutrition counselling  Prevention of hypoglycemia  Blood glucose monitoring | **n**  2  1  1  2  1  2  1  1  1 |  |  |

NICU: neonatal intensive care unit; VRIII: variable rate intravenous insulin infusion

**Table 3. Chronic hypertension** – scope of recommendations across 10 clinical practice guidelines

|  | **Screening** | | **Clinical interventions** | | | | **Health systems** | |
| --- | --- | --- | --- | --- | --- | --- | --- | --- |
| **Antenatal** | **Diagnosis of chronic hypertension**  Definitions and cut-off values  **Testing and monitoring**  Accurate dating of pregnancy  Baseline evaluation  Blood glucose  Proteinuria/creatine ratio  **Appointment frequency schedule** | **n**  7  1  2  1  5  1 | **Pharmacological therapy**  Antihypertensive therapy  Low-dose aspirin  **Treatment**  Acute-onset severe hypertension with antihypertensives  Superimposed pre-eclampsia with expectant management | **n**  7  2  1  1 | **Blood pressure monitoring and targets**  Self-monitoring  Blood pressure targets  **Obstetric care**  Fetal growth  Fetal wellbeing  **Referral**  Cardiologist  **Birth planning**  Timing of delivery | **n**  3  7  5  4  1  5 | **Health facility**  Staffing (Gynecologists)  Staffing (Obstetrician)  Equipment availability | **n**  1  1  1 |
| **Intrapartum** | No recommendations identified | | **Birth**  Mode of delivery  Antihypertensive therapy  Oxytocic | | | **n**  1  1  1 |  |  |
| **Postnatal** | **Monitoring immediately after delivery**  Blood pressure  **Follow-up**  Annual screening | **n**  1  1 | **Advice**  Weight management  **Blood pressure monitoring and targets**  Blood pressure monitoring  Blood pressure targets | | | **n**  2  2  1 |  |  |

**Table 4. Asthma** – scope of recommendations across 2 clinical practice guidelines

|  | **Screening** | | **Clinical interventions** | | | | **Health systems** |
| --- | --- | --- | --- | --- | --- | --- | --- |
| **Antenatal** | **Repeat testing**  Spirometry | **n**  1 | **Advice/Education**  Asthma management  Peak flow meter  Reporting fetal activity  Smoking cessation  **Initial assessment**  Severity classification criteria  Previous asthma medication use  Influenza vaccination  Smoking status  **Routine care**  Monitoring severity and control  Frequency of appointments  Medication review  Check inhaler technique  **Pharmacological therapy**  Inhaled glucocorticoids  ICS **±** LABA  Individualized care | **n**  1  1  1  1  1  1  1  1  1  1  1  1  1  1  1 | **Acute exacerbation management**  Pharmacological treatment  Monitoring  Lung function  Oxygen saturation  Fetal monitoring  **Moderate/severe or poorly controlled asthma management**  Monitoring  Fetal ultrasound  Chest X-ray  Pharmacological therapy  Frequency of appointments  Referral: anesthetic review  Referral: pulmonary medicine expert  Referral: obstetric/respiratory physician  **Management of co-morbidities**  **Birth planning**  Mode of delivery  Timing of delivery | **n**  1  1  1  1  1  1  1  1  1  1  1  1  1  1  1  1 | No recommendations identified |
| **Intrapartum** | No recommendations identified | | **Birth**  Induction of labor  Pain management  Pharmacological therapy  Hydration management | **n**  2  2  1  1 | **Acute exacerbations**  Clinical diagnosis criteria  Pharmacological management  Non-pharmacological management | **n**  1  1  1 |  |
| **Postnatal** |  |  | **Advice/education**  Smoking cessation  **Breastfeeding** | | | **n**  1  1 |  |

ICS: inhaled corticosteroids; LABA: long-acting beta2-agonists

**Table 5. Sickle cell disorder** – scope of recommendations across 1 clinical practice guideline

|  | **Screening** | **Clinical interventions** | | **Health systems** |
| --- | --- | --- | --- | --- |
| **Antenatal** | **Tests and screening**  Partner testing  Baseline renal function test  Urine protein/creatinine ratio  Midstream urine culture  Liver function test  Ferritin  Oxygen saturation  Blood pressure  Viability scan  End organ damage | **Advice/education**  Vaccinations  Avoiding precipitating factors  **Routine care**  Clinical history assessment  Medication review  Management of complications  Retinal, renal, cardiac assessment  Review by midwife  **Pharmacological therapy**  Analgesia  Low-dose aspirin  Low-molecular weight heparin  **Supplements**  Folic acid  **Blood transfusion**  Blood matching | **Acute pain management**  Analgesia  Thromboprophylaxis  Pharmacological therapy  Pain  Sedation  Antipruritic  Antiemetic  Home care and outpatient follow-up  **Birth planning**  Selection of hospital or clinic  Mode of delivery  Timing of delivery  Special consideration: hip replacements  Anesthetic assessment  **Obstetric care**  Fetal growth monitoring  Anomaly scan  Serial fetal biometry scan  Amniotic fluid volume monitoring | **Protocol**  Protocol for management of complications of sickle cell disease  **Health workforce**  Obstetrician, midwife, hematologist  Training of maternity care staff  **Auditing**  Medical records  **Referral pathway**  Specialist center or care network for shared care |
| **Intrapartum** | No recommendations identified | **Birth**  Temperature and fluid management  Analgesia  Electronic fetal heart rate monitoring | |  |
| **Postnatal** | **Postpartum screening**  Oxygen saturation  Level of hydration | **Pharmacological therapy**  Low-molecular-weight heparin | |  |

**Table 6. General mental disorders, bipolar disorder, psychotic disorders** – scope of recommendations across 6 clinical practice guidelines

|  | **Screening** | | **Clinical interventions** | | | | **Health systems** | |
| --- | --- | --- | --- | --- | --- | --- | --- | --- |
| **Antenatal** | **Assessment**  Family history of psychotic disorders and depressive disorders  Depressed and/or psychotic groups  Suicide and infanticide risk  Adolescent mothers - EPDS | **n**  2  1  1 | **General mood disorders**  Pharmacological treatment  Risk-benefit analysis  Electroconvulsive therapy  **Major mental illness**  Pregnancy and postnatal plan  Multidisciplinary team  **Bipolar disorder**  Individualized treatment plan  Remission stage  Pharmacological treatment  Antiepileptic drugs  Folic acid  Ultrasound  Psychosocial therapy  Electroconvulsive therapy  Referral: psychiatrist  Referral: genetic counselling | **n**  1  1  1  1  1  1  4  1  1  2  1  1  1 | **Psychotic disorders**  Individualized treatment plan  Pharmacological treatment  Fetal monitoring  Lithium management  Monitoring: mental state  **Schizophrenia**  Pharmacological treatment  Written informed consent  Risk-benefit analysis  Involuntary treatment  Monitoring: complications  Therapeutic drug monitoring  Supplementation: folate  Birth planning: type of facility  **Suicide and infanticide risk**  Involve other parties | **n**  1  2  1  1  1  11  1  1  1  1  1  1  1 | **Models of care**  National managed clinical network  **Bipolar disorder**  Integrated treatment plan  **Schizophrenia**  Multidisciplinary care: psychiatrists, gynecologists, pediatricians, midwives | **n**  1  1  1 |
| **Intrapartum** | No recommendations identified | | | | | |  |  |
| **Postnatal** | **Assessment**  Diagnosis of postpartum psychosis  Depressive symptoms  Adolescent mothers - EPDS  **Monitoring**  Changes in mental state  Infant monitoring  **Long-term monitoring**  Schizophrenia: Long-term complications in children | 1  1  1  1  2  1 | **Breastfeeding**  Indications for breastfeeding  Special considerations | 1  1 | **Bipolar disorder**  Pharmacological treatment  Treatment plan  Referral: specialist psychiatric assessment  **Postpartum psychosis**  Pharmacological treatment | 3  2  1  1 |  |  |

EPDS: the Edinburgh Postnatal Depression Scale

**Table 7. Depression and anxiety** – scope of recommendations across 10 clinical practice guidelines

|  | **Screening** | | **Clinical interventions** | | | | **Health systems** | |
| --- | --- | --- | --- | --- | --- | --- | --- | --- |
| **Antenatal** | **Assessment of depression**  Timing of assessment  Emotional wellbeing  Routine for high-risk women  Language and culturally appropriate tools  Ultrasound in second trimester  Provider: Obstetrician-gynecologists and others  **Psychosocial assessment (depression)**  Comprehensive assessment  Psychosocial factors  EPDS  EPDS + ANRQ  Whooley questions  Mode of delivery  **Assessment (Anxiety)**  Risk factors, direct observations  Emotional wellbeing  Ultrasound in second trimester | **n**  4  1  1  1  1  1  1  1  4  1  1  1  1  1  1 | **Education and advice**  Self-care strategies  Peer support  Lifestyle advice and sleep  **Depression: Management**  Comprehensive care  Mother-infant relationship  Treatment by severity  Home visits  **Depression: Pharmacotherapy**  Advise associated risks  Discontinuation of treatment  Medication review by GP  Risk-benefit analysis  Dosage adjustment  Benzodiazepines  SSRIs, SNRIs | **n**  2  2  3  1  1  1  1  1  2  1  1  2  1  1  3 | **Depression: Psychotherapy**  Shared decision-making  Cognitive behavioral therapy  Interpersonal psychotherapy  Psychodynamic therapy  Psychoeducation  **High-risk management**  Suicidal ideation  Severe depression  **Depression: Referral**  Mental health services  Counselling/Psychiatrist  **Depression: Birth planning**  Place of delivery  **Management of anxiety**  Information about mental health  Advice on lifestyle and sleep  Benzodiazepines  Folic acid supplementation | **n**  1  2  3  1  2  2  1  2  2  1  1  1  1  1 | **Guidelines**  Local guidelines for application of EPDS and ANRQ  Locally relevant strategies for appropriate, culturally responsive care  **Health provider competencies**  **Model of care**  Comprehensive and coordinated care  **Service availability**  Follow-up for diagnosis and treatment  **Education and training**  Tertiary education curriculum  Professional development  Woman-centered communication skills  Psychosocial assessment  Registered practitioners to have accredited training in the relevant therapy | **n**  1  1  4  1  1  1  1  1  1  1 |
| **Intrapartum** | No recommendations identified | | **Birth**  Pharmacotherapy | | | **n**  1 |  |  |
| **Postnatal** | **Assessment of depression**  Timing of assessment  Emotional wellbeing  Language and culturally appropriate tools  **Psychosocial assessment (depression)**  Comprehensive assessment  Mood and emotional wellbeing  EPDS  EPDS + ANRQ  Mode of delivery  **Assessment (Anxiety)**  Risk factors, direct observations  EPDS  Emotional wellbeing  **Neonatal assessment**  Risk of harm to infant  Adverse effects  Neonatal Adaptation Syndrome | **n**  5  1  1  1  1  4  1  1  1  1  1  2  1  1 | **Depression: Education and advice**  Self-care strategies  Peer support  Lifestyle advice and sleep  **Management**  Comprehensive care  Mother-infant relationship  Home visits  **Treatment by severity**  **Pharmacotherapy**  Risk-benefit analysis  Drug and dosage selection  Discontinuation of treatment  Prophylactic treatment  Benzodiazepines  SSRI, TCAs  **Psychotherapy**  Shared decision-making  Cognitive behavioral therapy  Interpersonal psychotherapy  Psychodynamic therapy  Psychoeducation  **High-risk management**  Suicidal ideation  **Referral**  Counselling | **n**  1  2  2  1  1  1  3  1  2  1  1  1  2  1  3  2  1  1  2  1 | **Management of anxiety**  Emotional wellbeing  Management of insomnia  Pharmacotherapy  SSRIs and SNRIs  Benzodiazepines  Psychotherapy, self-care  **Breastfeeding**  Indication for breastfeeding  Risk-benefit analysis  Nonexclusive breastfeeding  Medication considerations  Referral: lactation consultant | **n**  1  1  1  2  1  3  2  1  1  1 |  |  |

ANRQ: antenatal risk questionnaire; EPDS: the Edinburgh Postnatal Depression Scale; GP: general practitioner; SNRI: norepinephrine reuptake inhibitors; SSRI: selective serotonin reuptake inhibitors; TCAs: tricyclic antidepressants

**Table 8. Substance use disorders** – scope of recommendations across 7 clinical practice guidelines

|  | **Screening** | | **Clinical interventions** | | | | **Health systems** | |
| --- | --- | --- | --- | --- | --- | --- | --- | --- |
| **Antenatal** | **Approach to screening**  Universal  **Screening tools**  4Ps screening tool  NIDA Quick Screen  CRAFFT  Verbal screening  Urine drug screening  **Routine assessment**  Current and previous drug use  At-risk of inhalant use  Partner alcohol or drug use  Maternal and fetal wellbeing  Mental health  **Comprehensive assessment**  **Special considerations**  Adolescent mothers  Informed consent before testing | **n**  2  2  2  2  1  1  3  1  1  1  1  1  1  1 | **Advice/Education**  Adverse effects  Birth and postnatal planning  Verbal and written format  Cessation/detoxification services  **Management**  Care plan  Chronic pain management  Discharge plan  Harm reduction approach  Dental infections  Provide interpreter if required  **Psychosocial therapy**  Brief intervention  **Birth planning**  Anesthesia  Analgesia  Central venous access  Timing and mode of delivery  Pain management  **Obstetric care**  Fetal growth  **Child protection**  Case meetings to establish agreed plan of care | **n**  2  1  1  1  1  1  1  1  1  1  1  1  1  1  1  1  1  1 | **Amphetamines**  Cessation/Reduction  Provide counselling and advise risks  Monitor mental health  **Benzodiazepines**  Long-acting benzodiazepines  **Cannabis**  Abstinence/reduction and advise risks  Psychosocial interventions  **Opioid use**  Indication for detoxification  Methadone program  Buprenorphine/naloxone  Supervised detoxification  Split dosing  Services for heroin-dependent mothers  Treatment for partners  **Psychostimulants**  Pharmacological treatment indication  **Referral**  Drug and alcohol treatment  Services for mental health problems  **Management of late presentations**  Hospitalization  Comprehensive assessment | **n**  1  1  1  2  2  1  3  2  1  1  1  1  1  1  2  1  1  1 | **Policy/Guidelines**  Guideline on stabilization and psychosocial management  Guidelines for split dosing  Guideline for neonatal care  Systematic communication strategies  **Multidisciplinary team**  Health professionals, social services, probation, and parole officer  **Training/education**  Serious mental disorders  Care for vulnerable populations  **Service delivery**  Continuity of care  Service availability  Partnership model  **Legal - Child protection**  Liaise with statutory child protection agency  **Culturally sensitive services** | **n**  1  1  1  1  1  1  1  1  1  1  1  1 |
| **Intrapartum** | No recommendations identified | | **Birth**  Pain management  Anesthesia  Management of late presentation during labor | **n**  1  1  1 | **Opioid use**  Management of late presentations  Anesthesia  Management of buprenorphine users  Management of methadone users | **n**  1  1  1  1 |  |  |
| **Postnatal** | **Assessment**  Comprehensive assessment  Risk to infant  **Neonatal monitoring**  Neonatal abstinence syndrome  Finnegan Scale  Central nervous system depression | **n**  1  1  2  1  1 | **Advice/Education**  Neonatal abstinence syndrome  SIDS precautions  Safe sleeping practices  Skin-to-skin contact  Culturally appropriate education  **Management**  Pain management  Hospital admission  Discharge plan  Assess for home-visits  Follow-up with inpatient services | **n**  2  1  1  1  1  1  1  1  1  1 | **Benzodiazepines**  Pharmacological treatment  Phenobarbitone  Breastfeeding precautions  **Opioid use**  Review maintenance dose  Infant extended follow-up  Multiagency collaboration  **Cannabis**  Supportive settling techniques  **Breastfeeding**  Support or advise against  Advice for continuing users  Benzodiazepines  Opioids  Psychostimulants | **n**  1  1  1  1  1  1  1  2  1  1  3  1 |  |  |

NIDA: National Institute on Drug Abuse; SIDS: sudden infant death syndrome

**Table 9. Tobacco use** – scope of recommendations across 2 clinical practice guidelines

|  | **Screening** | | **Clinical interventions** | | | | **Health systems** | |
| --- | --- | --- | --- | --- | --- | --- | --- | --- |
| **Antenatal** | **Assessment**  Smoking status (universal)  Environmental tobacco smoke  **Identified smokers**  Comprehensive assessment  Test for nicotine dependence | **n**  1  1  1  1 | **Advice/Education**  Smoking cessation  Harmful effects of smoking  Passive smoking  Culturally sensitive counselling  **Frequency of support**  Routine visit  **Management**  Relapse prevention | **n**  1  1  1  1  1  1 | **Smoking cessation**  Brief intervention approach  5As  Psychosocial interventions  Pharmacological therapy  Nicotine replacement therapy  Special considerations  Antipsychotic use  Support for partner | **n**  1  2  1  2  1  1 | **Models of care**  Integrated services  **Referral pathways**  Relapse prevention | **n**  1  1 |
| **Intrapartum** | No recommendations identified | | | | | |  |  |
| **Postnatal** | **Assessment**  Smoking status  Environmental tobacco smoke | **n**  1  1 | **Breastfeeding**  Risk assessment  Nicotine replacement therapy  **Education and advice**  Risks of smoking  Environmental tobacco smoke | | | **n**  1  1  1  1 |  |  |

**Table 10. Alcohol use disorder** – scope of recommendations across 6 clinical practice guidelines

|  | **Screening** | | **Clinical interventions** | | | | **Health systems** | |
| --- | --- | --- | --- | --- | --- | --- | --- | --- |
| **Antenatal** | **Approach to screening**  Universal screening  Routine alcohol use screening  **Assessment**  Low/moderate/high risk  Comprehensive assessment  Substance use  **Screening tools**  Drink measure  AUDIT-C  T-ACE  TWEAK  Self-report | **n**  3  1  1  1  1  1  3  4  2  2 | **Advice/education**  **Cessation/reduction of alcohol intake**  Risks of alcohol use during pregnancy  Alcohol toxicity  Provide educational materials  Educate father/partners  **Management**  Harm reduction  Individualized & comprehensive care  By severity of illness  Social support  **Pharmacological therapy**  Risk-benefit analysis  Indication for pharmacological therapy  Disulfiram – with caution  **Psychosocial therapy**  Brief intervention  5As Model | **n**  3  1  1  1  1  2  3  1  1  1  1  1  1  5  1 | **Treatment - acute withdrawal**  Assessment  CIWA-R  PAWSS  **Medically assisted withdrawal**  Benzodiazepines  Naltrexone  Nutrition and vitamin supplementation  Maternal and fetal monitoring  Hospitalization/ Inpatient setting  Continuing AUD care  **Treatment – Alcohol detoxification**  Hospitalization  **Referral**  Drug and alcohol specialist | **n**  1  1  5  2  1  2  1  2  1  1  2 | **Policies**  Aligned to promote support and treatment by providers  **Multidisciplinary team**  **Health provider training**  AUD treatment  Culturally sensitive drinking habits  **Service delivery**  Referral pathways  Community-based interventions  Comprehensive medical care  **Environment**  Sensitive to gender and cultural issues  Non-judgmental and supportive  Respectful and collaborative therapeutic relationship  Incorporate principles of trauma- and violence-informed care | **n**  1  1  1  1  1  1  1  1  1  1  1  1 |
| **Intrapartum** | **Assessment**  Withdrawal, intoxication symptoms | **n**  1 | No recommendations identified | | | |  |  |
| **Postnatal** | **Routine screening**  Alcohol use  **FASD neonate assessment**  Meconium testing - EtG, FAEEs  **Neonatal monitoring**  Benzodiazepine effects | **n**  1  4  1  1 | **Advice**  Rooming in, skin-to-skin contact  **Psychosocial therapy**  Counselling, Brief intervention  **Management**  Mother/baby contact  Prolonged hospitalization if required  Neonates with FASD  Withdrawal management | **n**  1  1  1  1  1  1 | **Breastfeeding**  Cessation/Reduction  Advice for alcohol consumption  Pharmacotherapy indication  **Referral**  Pediatricians  Psychiatrists  Infants/young children -  trained health professional | **n**  4  1  1  1  1  1 |  |  |

AUD: alcohol use disorder; AUDIT-C: alcohol use disorders identification test; CIWA-R: clinical institute withdrawal assessment of alcohol scale, revised; FAEE: meconium fatty acid ethyl esters; FASD: fetal alcohol spectrum disorder; PAWSS: post-acute withdrawal syndrome
